# Pharmacological Dissection Identifies Retatrutide Overcomes the Therapeutic Barrier of Obese TNBC Treatments through Suppressing the Interplay between Glycosylation and Ubiquitylation of YAP

**DOI:** 10.1002/advs.202407494

**Published:** 2025-01-27

**Authors:** Xin Cui, Yueming Zhu, Lidan Zeng, Mengyuan Zhang, Amad Uddin, Theresa W. Gillespie, Lauren E. McCullough, Shaying Zhao, Mylin A. Torres, Yong Wan

**Affiliations:** ^1^ Department of Pharmacology and Chemical Biology Emory University School of Medicine Atlanta GA 30322 USA; ^2^ Winship Cancer Institute Emory University School of Medicine Atlanta GA 30322 USA; ^3^ Department of Biochemistry and Molecular Biology Institute of Bioinformatics University of Georgia Athens GA 30602 USA; ^4^ Department of Surgery Emory University School of Medicine Atlanta GA 30322 USA; ^5^ Department of Hematology and Medical Oncology Emory University School of Medicine Atlanta GA 30322 USA; ^6^ Department of Epidemiology Emory University Rollins School of Public Health Atlanta GA 30322 USA; ^7^ Department of Radiation Oncology Emory University School of Medicine Atlanta GA 30322 USA

**Keywords:** adipocyte, deubiquitinase, glycosylation, OGT, tumorigenesis, YAP

## Abstract

Triple‐negative breast cancer (TNBC) in obese patients remains challenging. Recent studies have linked obesity to an increased risk of TNBC and malignancies. Through multiomic analysis and experimental validation, a dysfunctional Eukaryotic Translation Initiation Factor 3 Subunit H (EIF3H)/Yes‐associated protein (YAP) proteolytic axis is identified as a pivotal junction mediating the interplay between cancer‐associated adipocytes and the response to anti‐cancer drugs in TNBC. Mechanistically, cancer‐associated adipocytes drive metabolic reprogramming resulting in an upregulated hexosamine biosynthetic pathway (HBP). This aberrant upregulation of HBP promotes YAP O‐GlcNAcylation and the subsequent recruitment of EIF3H deubiquitinase, which stabilizes YAP, thus promoting tumor growth and chemotherapy resistance. It is found that Retatrutide, an anti‐obesity agent, inhibits HBP and YAP O‐GlcNAcylation leading to increased YAP degradation through the deprivation of EIF3H‐mediated deubiquitylation of YAP. In preclinical models of obese TNBC, Retatrutide downregulates HBP, decreases YAP protein levels, and consequently decreases tumor size and enhances chemotherapy efficacy. This effect is particularly pronounced in obese mice bearing TNBC tumors. Overall, these findings reveal a critical interplay between adipocyte‐mediated metabolic reprogramming and EIF3H‐mediated YAP proteolytic control, offering promising therapeutic strategies to mitigate the adverse effects of obesity on TNBC progression.

## Graphical abstract

This study reveals that obesity‐driven metabolic dysfunction enhances YAP O‐GlcNAcylation, facilitating its stabilization by preventing ubiquitylation and degradation via EIF3H, thereby driving TNBC progression and chemoresistance. Retatrutide disrupts this axis by suppressing O‐GlcNAcylation, restoring YAP ubiquitylation, and enhancing degradation, sensitizing TNBC cells to chemotherapy. These findings highlight the therapeutic potential of targeting glycosylation‐ubiquitylation crosstalk in TNBC.

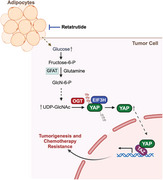

## 1 Introduction

Triple‐negative breast cancer (TNBC) remains one of the most challenging subtypes of breast cancer due to its aggressive nature, lack of targeted therapies, and poor prognosis.^[^ ^1^, ^2^ ^]^ It is well‐established that obesity is a modifiable risk factor contributing to the incidence and progression of various cancers.^[^ ^3^, ^4^, ^5^, ^6^, ^7^, ^8^, ^9^, ^10^ ^]^ While prior studies have predominantly concentrated on the association between obesity and luminal breast cancer carcinogenesis and the resistance to endocrine therapy,^[^ ^11^, ^12^, ^13^, ^14^ ^]^ there is an inadequacy of comprehensive research addressing the role of obesity in the context of TNBC. The pathophysiological mechanisms by which obesity may influence TNBC development and progression are multifaceted.^[^ ^15^, ^16^, ^17^, ^18^ ^]^ The obese state is not merely a passive condition but actively contributes to tumor pathogenesis by creating an environment that favors cancer progression and resistance to therapy.^[^ ^19^ ^]^ Thus, there is an urgent need to understand the underlying molecular mechanisms that link obesity with TNBC pathogenesis and treatment resistance.

Recent advances in multi‐omic technologies have provided unprecedented insights into the complex biological networks that govern cancer cell behavior in the context of an obese microenvironment.^[^ ^20^, ^21^ ^]^ A central theme emerging from these studies is the significant role of tumor cell metabolic reprogramming driven by cancer‐associated adipocytes (CAAs).^[^ ^22^, ^23^ ^]^ One of the notable metabolic pathways affected by obesity is the hexosamine biosynthetic pathway (HBP).^[^ ^24^ ^]^ The HBP is responsive to the availability of nutrients and energy status and is critical for the production of UDP‐N‐acetylglucosamine (UDP‐GlcNAc),^[^ ^25^, ^26^ ^]^ which is essential for the post‐translational modification O‐GlcNAcylation of proteins.^[^ ^27^, ^28^ ^]^ O‐linked N‐acetylglucosamine (GlcNAc) transferase (OGT) is the enzyme responsible for catalyzing O‐GlcNAcylation, a dynamic and reversible post‐translational modification that adds N‐acetylglucosamine (GlcNAc) to serine and threonine residues of target proteins. This modification is highly sensitive to cellular metabolic states, as OGT activity is tightly linked to the hexosamine biosynthetic pathway (HBP), which integrates glucose and fatty acid metabolism. O‐GlcNAcylation affects a wide range of cellular processes, including protein stability, localization, interaction, and function. These modifications often act as molecular switches, competing with phosphorylation on the same or adjacent residues, thereby influencing signal transduction and cellular responses.^[^ ^29^, ^30^ ^]^ Notably, in TNBC, we previously found that an upregulated HBP has been associated with the hyperactivation of the oncogenic factor Yes‐associated protein (YAP).^[^ ^31^, ^32^ ^]^

The YAP protein is a transcriptional co‐activator involved in cell proliferation and apoptosis, and its deregulation has been implicated in various cancers, including TNBC.^[^ ^33^, ^34^, ^35^, ^36^, ^37^ ^]^ In TNBC, YAP overexpression or enhanced nuclear activity promotes tumorigenesis by regulating genes essential for cell proliferation and survival, which in turn supports tumor growth and metastasis.^[^ ^38^, ^39^ ^]^ Furthermore, YAP influences the breast cancer microenvironment by modulating tumor angiogenesis and immune evasion mechanisms.^[^ ^40^, ^41^, ^42^, ^43^, ^44^ ^]^ Crucially, the activation of YAP contributes to resistance against conventional treatments like chemotherapy and targeted therapies.^[^ ^45^, ^46^, ^47^, ^48^ ^]^ This resistance occurs through the support of cancer stem cell populations and modifications in drug transport mechanisms, presenting a substantial challenge in TNBC treatment strategies.^[^ ^49^ ^]^ In this study, through a comprehensive multiomic analysis, we have uncovered a novel metabolic reprogramming in TNBC driven by cancer‐associated adipocytes (CAAs). Under normal conditions, YAP is tightly regulated by a complex proteolytic system, which ensures its timely degradation and prevents aberrant cell growth. However, in the obese TNBC setting, the presence of CAAs leads to increased HBP activity, resulting in enhanced O‐GlcNAcylation of YAP. This modification shields YAP from proteasomal degradation by recruiting the deubiquitinase Eukaryotic Translation Initiation Factor 3 Subunit H (EIF3H), leading to YAP stabilization, tumor growth, and diminished chemotherapy effectiveness. Previous strategies targeting YAP have included inhibiting its activation, disrupting crucial YAP‐transcriptional enhanced associate domain (TEAD) interactions for gene transcription, and modulating its localization to prevent nuclear activity.^[^ ^50^ ^]^ Compounds like Verteporfin and Dasatinib have demonstrated efficacy in this context.^[^ ^51^, ^52^ ^]^ These efforts highlight progress in addressing YAP's complex regulation and its essential physiological roles. However, challenges persist due to YAP's dual roles in both physiological processes and disease states, requiring more selective therapeutic approaches. In the pursuit of innovative therapeutic strategies, by pharmacologically targeting the HBP/EIF3H/YAP axis, we demonstrate the feasibility of reactivating the degradation process of YAP, thereby enhancing the potency of chemotherapeutic agents and impeding tumor progression. Notably, in our study, the application of anti‐obesity agents such as Retatrutide in preclinical models of obese TNBC demonstrated significant inhibition of the HBP, leading to decreased levels of YAP. This downregulation of YAP is associated with an increase in the effectiveness of chemotherapy and a reduction in tumor volume, particularly in obese mouse models.

Our study highlights a critical intersection between the metabolic reprogramming by adipocytes and the proteolytic regulation of EIF3H‐YAP, offering insights into their combined impact on the progression and therapy resistance of TNBC in the context of obesity. By deepening our understanding of this interaction, we identify a promising therapeutic strategy that not only targets the oncogenic processes prevalent in TNBC but also leverages metabolic interventions. This dual approach could enhance existing cancer therapies and improve the prognosis for patients with TNBC affected by obesity, suggesting a multifaceted treatment pathway that addresses both oncogenic and metabolic challenges.

## 2 Results

### 2.1 Enhanced Hippo‐YAP Signaling Associates with Obese Breast Cancer Patients

**Figure 1:**
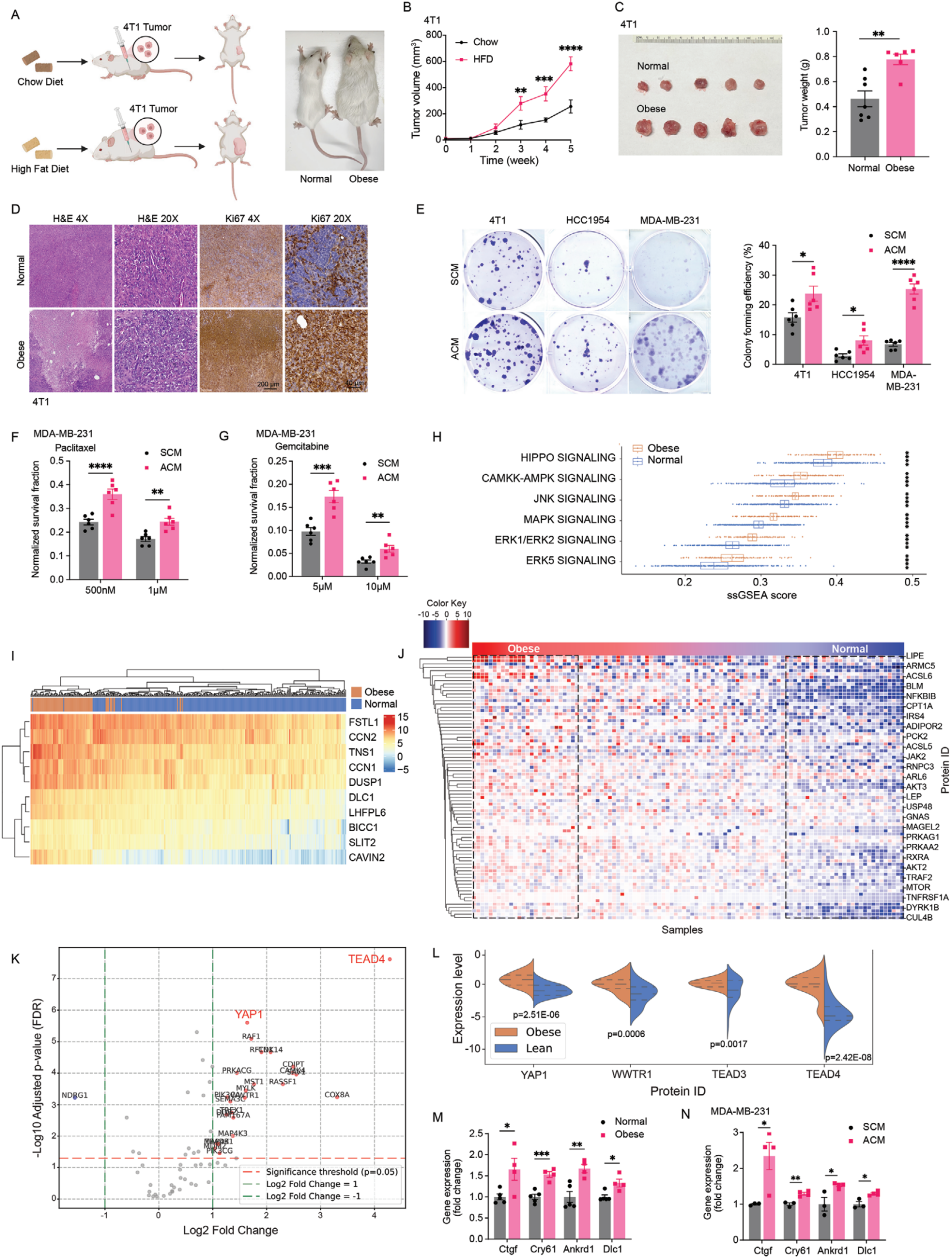
Hippo‐YAP signaling correlates with adipocyte‐initiated breast tumor carcinogenesis and suppresses therapeutic efficacy. A–C) Schematic diagram of eight‐week‐old Female BALB/c mice were fed with a normal chow diet or high‐fat diet (HFD) for 10 weeks. 4T1 breast cancer cells were orthotopically injected into the right fourth mammary gland of the mice (A). Tumor growth curve (B) was plotted (*n* = 5), and tumor weight (C) was measured at the endpoint (Normal *n* = 7, Obese *n* = 6). D) Each tumor was fixed in 4% buffered formaldehyde, paraffin‐embedded, and processed for hematoxylin‐eosin (H&E) staining and Ki67 staining. E) Stimulation of TNBC cell proliferation by adipocyte‐conditioned medium at low density as determined by colony formation assay. Represented figures were presented, and colony formation ratios were calculated (*n* = 6). F,G) Clonogenic cell survival experiments for ACM/SCM treated MDA‐MB‐231 cells with or without exposure to 500 nm/1 µm Paclitaxel (F) and 5 µm/10 µm Gemcitabine (G) for 24h, as measured by cytotoxicity (*n* = 6). H) Comparison of ssGSEA score distributions between obese and normal samples, focusing on tumor proliferation and survival pathways in breast tumors. P‐values are derived from Wilcoxon tests. I) Heatmap presents the row‐scaled log2(counts) values of 10 YAP‐conserved genes from obese and normal samples, ordered from left to right by expression level from high (obese) to low (normal). J) Heatmap and hierarchical clustering display the row‐scaled log2 protein expression values of 79 obesity‐associated genes from 105 human breast tumors (columns), arranged from left to right by expression level from high (obese) to low (normal). The log2 relative gene expression scale is depicted on the top left. K) Volcano plots showing the cancer hallmark gene expression changes focusing on tumor proliferation and survival pathways in obese breast cancer patient cohorts. Each circle represents one protein. The log fold change is represented on the x‐axis. The y‐axis shows the FDR adjusted log10 of the p‐value. A p‐value of 0.05 and a fold change of 1 are indicated by red and green lines. L) YAP, TEAD3/4, and WWTR1 expression density in obese and lean breast cancer patients were calculated based on log2 relative protein expression and represented in a violin plot. M,N) Increased Hippo‐YAP signaling pathway activation in obese mice bearing tumor (M) and adipocyte‐conditioned medium cultured MDA‐MB‐231 cells (N) as determined by RT‐PCR (Normal *n* = 5, Obese *n* = 4; SCM *n* = 3, ACM *n* = 4). Data (mean ± SEM) are representative of at least three independent experiments. *p < 0.05, **p < 0.01, ***p < 0.001, and ****p < 0.0001, by multiple unpaired T‐test.

To investigate the impact of obesity on breast cancer progression and treatment efficacy, we developed a diet‐induced obesity model in female BALB/c mice, achieved by subjecting the mice to a high fat diet (HFD) or maintaining them on a normal chow diet (Chow) for 10 weeks. We observed a marked increase in body weight and mammary fat pad weight in the HFD group when compared to their lean counterparts (Figure S1A–C, Supporting Information). Next, we orthotopically implanted 4T1 breast cancer cells into the right fourth mammary gland. Notably, the obese mice exhibited significantly accelerated tumor growth and increased tumor mass relative to the normal group (**Figure** **1**A–C). Histological analyses of the excised tumors revealed higher Ki67 expression in the obese mice, indicative of an enhanced proliferative rate within adipose‐enriched microenvironment. (Figure 1D). To further dissect the role of adipocytes in cancer progression, we utilized a co‐culture system simulating the tumor‐adipocyte interplay. Adipocyte conditioned medium (ACM) markedly increased colony formation in several TNBC cell lines, including 4T1, HCC1954, and MDA‐MB‐231. Notably, this proliferative effect was detectable following a brief 24 h exposure to ACM (Figure 1E). Additionally, we treated ACM‐ or stromal cell conditioned medium (SCM)‐preconditioned MDA‐MB‐231 and 4T1 cells with various chemotherapeutic agents, such as Carboplatin, Gemcitabine, and Paclitaxel to determine the adipocyte‐mediated modulatory effects on breast cancer treatment responses. The resulting clonogenic assays exhibited differential resistance patterns, with ACM‐pretreated cells displaying enhanced survival (Figure 1F,G; Figure S1D, Supporting Information), the proliferation analysis in MDA‐MB‐231 and 4T1 cells showed enhanced cell growth (Figure S1E–H, Supporting Information), suggesting that obesity may confer a protective effect against chemotherapy‐induced cytotoxicity.

To elucidate the mechanisms by which obesity influences tumor progression, we conducted comprehensive mRNA and proteomic analyses using data from The Cancer Genome Atlas (TCGA) and Clinical Proteomic Tumor Analysis Consortium (CPTAC) databases. By categorizing breast cancer patients into normal and obese groups based on their obesity signature gene expression profiles (Figure S2A, Supporting Information), our analysis revealed several dysregulated pathways, notably the Hippo‐YAP signaling pathway, which was the most significantly upregulated in the obese group (Figure 1H). While the c‐Jun N‐terminal kinases (JNK) and mitogen‐activated protein kinase (MAPK) pathways also exhibit distinct activation patterns between the normal and obese groups, as observed in our ssGSEA analysis (Figure 1H), our study primarily focuses on the Hippo‐YAP signaling pathway due to its significant alteration and its direct involvement in obesity‐driven tumor progression. The Hippo‐YAP pathway demonstrated the highest degree of alteration in terms of ssGSEA scoring, highlighting its central role in mediating metabolic reprogramming and tumor‐promoting functions in the obese tumor microenvironment. Further investigation showed elevated expression of key downstream genes in the Hippo‐YAP pathway associated with tumor proliferation and survival in obese patients, as demonstrated through both heatmaps and boxplots (Figure 1I; Figure S2B, Supporting Information). In addition, the same obesity signature gene set shown in Figure S2A (Supporting Information) was used to stratify breast cancer patients into obese and normal groups based on proteomic data from 105 breast cancer patients (Figure 1J; Figure S3, Supporting Information). Volcano plots comparing several selected key dysregulated pathways, including Hippo‐YAP, AMP‐activated protein kinase (AMPK), JNK, MAPK, and extracellular‐regulated kinase (ERK), revealed that YAP‐TEAD‐related proteins were significantly increased (Figure 1K). Additionally, we quantified the expression differences in key YAP‐related proteins, such as YAP, TEAD3/4, and WW Domain Containing Transcription Regulator 1 (WWTR1). These proteins were significantly upregulated in obese patients (Figure 1L). To validate these bioinformatic findings, we performed RT‐PCR to examine Hippo‐YAP pathway activity in an obese mouse model and in vitro co‐culture systems that mimic an obesity‐rich microenvironment (Figure 1M,N). The results confirmed a significant upregulation of Hippo‐YAP signaling downstream genes in both in vivo and in vitro obese settings, supporting the hypothesis that YAP may play a crucial role in linking obesity with adverse breast cancer outcomes.

### 2.2 Hippo‐YAP Signaling Axis as a Critical Mediator in the Complex Interplay between Obesity and Breast Cancer Pathogenesis

**Figure 2:**
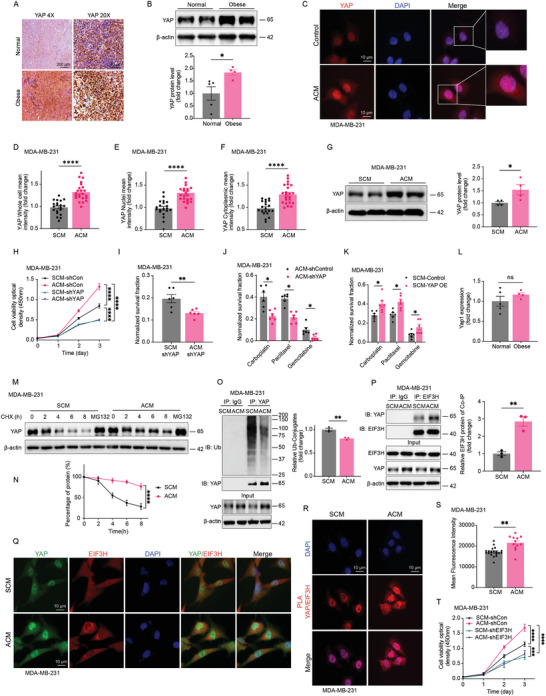
Adipocyte‐associated signaling promotes TNBC malignancy through proteolytic regulation of YAP stabilization. A) Tumor xenografts from obese and lean mice were immunohistochemically stained for YAP. B) YAP protein level was determined and quantified in obese and lean mice bearing tumors (Normal *n* = 5, Obese *n* = 4). C–F) Immunofluorescence staining of YAP protein expression in ACM and SCM treated MDA‐MB‐231. Scale bar = 10 µm. Whole cell, nuclei, and cytoplasmic YAP fluorescence intensities were quantified (D‐F) (SCM *n* = 20, ACM *n* = 23). G) YAP protein level was determined and quantified in ACM and SCM treated MDA‐MB‐231 breast cancer cells (*n* = 4). H) MDA‐MB‐231‐shCon and MDA‐MB‐231‐shYAP cells were cultured using adipocytes conditioned medium or stromal‐cell conditioned medium, cell proliferation rate was determined and quantified (*n* = 4). I) MDA‐MB‐231‐shCon and MDA‐MB‐231‐shYAP cells were pre‐treated with cultured using adipocytes conditioned medium or stromal‐cell conditioned medium for 24 h, and colony formations were determined (*n* = 6). J,K) Adipocytes conditioned medium cultured MDA‐MB‐231‐shYAP (J) and MDA‐MB‐231‐YAP OE (K) cells were treated with 25 µg ml^−1^ carboplatin, 500 nm paclitaxel and 5 µm gemcitabine for 24 h, and colony formations were determined (*n* = 6). L) YAP mRNA levels were determined by RT‐PCR in normal and obese mice bearing tumors (Normal *n* = 5, Obese *n* = 4). M,N) MDA‐MB‐231 cells were treated with cycloheximide or MG132, and YAP protein levels were determined (M) and quantified (N) (*n* = 3). O) Validation of YAP ubiquitylation by co‐immunoprecipitation of endogenous YAP in both ACM and SCM‐treated MDA‐MB‐231 cells (*n* = 3). P) Validation of EIF3H‐YAP interaction by co‐immunoprecipitation of endogenous EIF3H in both ACM and SCM‐treated MDA‐MB‐231 cells. The YAP‐associated EIF3H was quantified on the right (*n* = 3). Q) Colocalization between YAP and EIF3H and DAPI was measured by immunofluorescence staining, showing a principal overlap of YAP (green) and EIF3H (red) in both cytosol and nuclei. R,S) Slides of SCM or ACM treated MDA‐MB‐231 cells were incubated with mouse anti‐YAP and rabbit anti‐EIF3H antibodies. Duolink PLA was then performed and red dots indicate the interaction of the two proteins (R). The fluorescence intensities were quantified in (S) (SCM *n* = 19, ACM *n* = 12). T) MDA‐MB‐231‐shCon and MDA‐MB‐231‐shEIF3H cells were cultured using adipocytes conditioned medium or stromal‐cell conditioned medium, cell proliferation rate was determined and quantified (*n* = 4). Data (mean ± SEM) are representative of at least three independent experiments. *p < 0.05, **p < 0.01, ***p < 0.001, and ****p < 0.0001, by multiple unpaired t‐test or two‐way ANOVA followed with Tukey's multiple comparisons test.

To explore the role of YAP signaling in adipocyte‐driven breast tumor carcinogenesis and its impact on treatment response, we analyzed tumor xenografts from obese and lean mice. Immunohistochemical (IHC) staining and immunoblotting of tumor xenografts from obese and lean mice demonstrated markedly elevated YAP expression in the tumors derived from obese mice (**Figure** **2**A,B). This finding was mirrored in our in vitro studies, where MDA‐MB‐231 cells cultured with adipocyte‐conditioned medium (ACM) exhibited marked increases in YAP protein levels, as demonstrated by immunofluorescence and immunoblotting, relative to those treated with stromal‐cell conditioned medium (SCM) (Figure 2C–G). Next, we evaluated the proliferation and colony formation abilities in both control and YAP‐shRNA‐silenced (shYAP) MDA‐MB‐231 and 4T1 cell lines exposed to ACM or SCM. The results (Figure 2H,I; Figure S4A,B, Supporting Information) demonstrated that shYAP significantly decreased proliferation and colony formation in both cell lines, with a more pronounced inhibitory effect observed in the ACM‐treated groups. These findings underscore the critical role of YAP in mediating the pro‐tumorigenic effects of ACM. In contrast, 4T1 and MDA‐MB‐231 cells with YAP overexpression further promoted ACM‐induced cell proliferation (Figure S4C–F). This suggests that YAP plays a key role in the adipocyte‐enhanced growth of cancer cells. Notably, YAP downregulation also sensitized ACM‐treated MDA‐MB‐231 breast cancer cells to multiple cytotoxic agents. (Figure 2J). In contrast, SCM pre‐treated TNBC cells overexpressed YAP displayed increased resistance to such treatments (Figure 2K). These findings collectively underscore the pivotal role of YAP signaling in the mechanism of adipocyte‐mediated tumor progression and the modulation of therapeutic efficacy.

To identify the potential mechanisms through which obesity‐related signaling might influence the upregulation of YAP expression, we initially utilized RT‐PCR to determine whether this signaling could directly alter YAP transcription. Contrary to expectations, our RT‐PCR analysis did not reveal any significant changes in YAP transcription levels in either obese mice or ACM‐treated MDA‐MB‐231 cells (Figure 2L; Figure S4G, Supporting Information). We then shifted our focus to post‐translational modifications, specifically the stability of the YAP protein, which is known to be intricately regulated.^[^ ^53^, ^54^ ^]^ A pulse‐chase assay demonstrated that MDA‐MB‐231 and HCC1954 cells exposed to ACM exhibited a significantly slower YAP protein turnover rate compared to controls (Figure 2M,N; Figure S4H, Supporting Information). Subsequent co‐immunoprecipitation of the YAP protein complex showed reduced ubiquitylation in the presence of ACM (Figure 2O). This decrease in ubiquitin tagging indicates a shift toward enhanced stability of YAP in cells within an obese microenvironment. Prior studies have identified EIF3H as a critical deubiquitinase of YAP, utilizing a unique catalytic triad (Asp90, Asp91, and Gln121) and key residues (Trp119 and Tyr140) that interact with the N‐terminal region of YAP1 to form a stable complex, thereby promoting YAP deubiquitylation, stabilization, and transactivation.^[^ ^55^ ^]^ To further investigate EIF3H‐YAP interactions, we examined MDA‐MB‐231, HCC1954, and 4T1 cells cultured in adipocyte‐conditioned media (ACM). Immunoprecipitation assays (Figure 2P) indicated an increase in EIF3H‐YAP interactions upon ACM treatment. These results were further validated through immunofluorescence staining (Figure 2Q; Figure S4I,L, Supporting Information) and proximity ligation assays (PLA) (Figure 2R,S; Figure S4J,K,M,N, Supporting Information), which consistently showed enhanced EIF3H‐YAP interactions across all MDA‐MB‐231 (Figure 2Q–S, Supporting Information), HCC1954 (Figure S4I–K, Supporting Information) and 4T1 (Figure S4L–N, Supporting Information) cells treated with ACM. Interestingly, PLA assays revealed a significant increase in EIF3H‐YAP interactions in both the nucleus and cytosol of all cell lines. However, the interaction patterns differed between cell lines: MDA‐MB‐231 cells displayed predominantly nuclear interactions under ACM treatment, while 4T1 and HCC1954 cells exhibited stronger cytosolic interactions. These variations likely reflect cell line‐specific regulatory mechanisms or differences in Hippo pathway dynamics across TNBC subtypes. Furthermore, MDA‐MB‐231 cells with EIF3H knockdown mirrored the tumor cell growth and colony formation inhibition effects seen in (Figure 2T; Figure S4O–Q, Supporting Information). These observations confirm the critical role of the EIF3H‐YAP axis in mediating the adipocyte‐induced accumulation of YAP and the subsequent tumor promotion.

### 2.3 Interplay between Glycosylation and Ubiquitylation Dictates Adipocyte Signaling Induced YAP Accumulation through Metabolic Dysregulation

**Figure 3:**
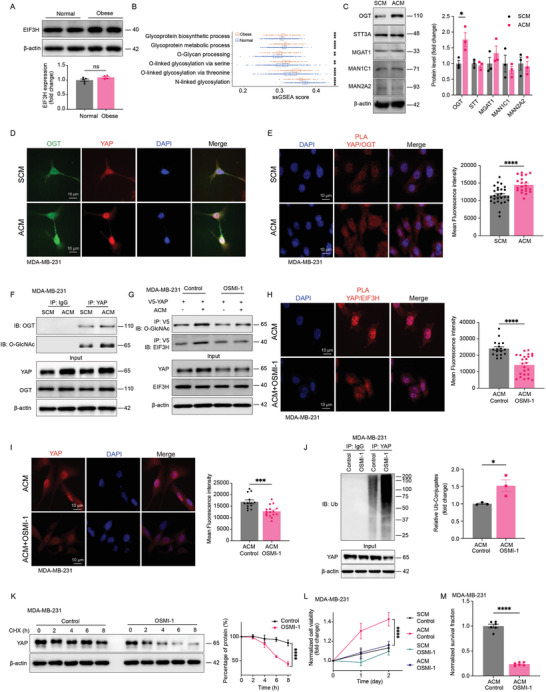
Interplay between glycosylation and ubiquitylation dictates adipocyte induced YAP accumulation. A) EIF3H protein level was determined and quantified in TNBC tumors from obese and normal mice (Normal *n* = 5, Obese *n* = 4). B) Comparison of ssGSEA score distributions between obese and normal samples in breast tumors focuses on glycosylation‐related signaling pathways. P‐values are obtained from Wilcoxon tests. C) The protein expression levels of multiple enzymes involved in both O‐glycosylation and N‐glycosylation pathways, including OGT, STT3A, MGAT1, MAN1C1, and MAN2A2, were determined in ACM or SCM treated MDA‐MB‐231 cells (*n* = 3). D) Colocalization between YAP and OGT and DAPI was detected by immunofluorescence staining, showing a principal overlap of OGT (green) and YAP (red) in the cytosol. E) Slides of SCM or ACM treated MDA‐MB‐231 cells were incubated with mouse anti‐YAP and rabbit anti‐OGT antibodies. Duolink PLA was then performed and red dots indicate the interaction of the two proteins. The red fluorescence was also quantified (SCM *n* = 27, ACM *n* = 21). F) Validation of biochemical interaction between YAP and OGT in ACM or SCM‐treated MDA‐MB‐231 cells by co‐immunoprecipitation of endogenous YAP. G) ACM‐conditioned MDA‐MB‐231 cells were treated with OSMI‐1, and both YAP O‐GlcNAcylation and the interaction between YAP and EIF3H were determined by co‐immunoprecipitation. H) ACM‐conditioned MDA‐MB‐231 cells were treated with OSMI‐1 and then incubated with mouse anti‐YAP and rabbit anti‐EIF3H antibodies. Duolink PLA was then performed and red dots indicate the interaction of the two proteins. The red fluorescence was also quantified (Control *n* = 18, OSMI‐1 *n* = 24). I) ACM‐conditioned MDA‐MB‐231 cells were treated with OSMI‐1 and Immunofluorescence staining of YAP expression (red) was measured and quantified (Control *n* = 13, OSMI‐1 *n* = 16). J) Validation of YAP ubiquitylation by co‐immunoprecipitation of endogenous YAP in OSMI‐1 treated ACM cultured MDA‐MB‐231 cells (*n* = 3). K) OSMI‐1 treated ACM conditioned MDA‐MB‐231 cells were incubated with cycloheximide and YAP protein turnover was determined (*n* = 3). L,M) MDA‐MB‐231 cells were cultured using adipocytes conditioned medium and treated with OSMI‐1 for 24 h, CCK8 based cell proliferation (L) (*n* = 4), and colony formation (M) (*n* = 6) were determined. Data (mean ± SEM) are representative of at least three independent experiments. *p < 0.05, **p < 0.01, ***p < 0.001, and ****p < 0.0001, by multiple unpaired t‐test or two‐way ANOVA followed with Tukey's multiple comparisons test. OGT, O‐linked N‐acetylglucosamine (GlcNAc) transferase. STT3A, STT3 oligosaccharyltransferase complex catalytic subunit A. MGAT1, alpha‐1,3‐mannosyl‐glycoprotein 2‐beta‐N‐acetylglucosaminyltransferase. MAN1C1, mannosidase alpha class 1C member 1. MAN2A2, mannosidase alpha class 2A member 2.

While we observed an increased interaction between EIF3H and YAP, there was no corresponding increase in EIF3H expression in obese mice or following treatment with adipocyte‐conditioned medium (**Figure** **3**A; Figure S5A, Supporting Information), suggesting an alternative mechanism that promotes the EIF3H‐YAP interaction. Ubiquitylation is known to be intricately regulated and often exhibits crosstalk with other PTM processes, and considering the established regulatory crosstalk between protein glycosylation and ubiquitylation that governs protein stability,^[^ ^56^ ^]^ we hypothesize that such mechanisms could be at play here. Additionally, Obesity‐related metabolic reprogramming is associated with aberrant glycan processing. Given this context, it is plausible that obesity‐induced metabolic reprogramming may alter YAP glycosylation patterns, which could, in turn, affect YAP ubiquitylation by modulating interactions with the deubiquitinase EIF3H.

To validate our hypothesis, we analyzed the ssGSEA score distributions for protein glycosylation pathways in breast cancer patients, comparing obese and normal weight groups. The results, presented in (Figure 3B), clearly demonstrated that obese breast cancer patients exhibit significantly higher levels of O‐glycan processing and subsequent O‐linked glycosylation. Next, we initiated a screening of key enzymes involved in both O‐glycosylation and N‐glycosylation pathways. The data presented in (Figure 3C) show a significant increase of O‐GlcNAc transferase (OGT) in MDA‐MB‐231 cells treated with adipocyte‐conditioned media (ACM), similar results were observed in 4T1 cells shown in (Figure S5B, Supporting Information). Further analysis via co‐immunoprecipitation, immunofluorescent staining, and PLA assay confirmed our findings, demonstrating an increased interaction between OGT and YAP, along with an increase in YAP O‐GlcNAcylation levels (Figure 3D–F).

Conversely, treatment of MDA‐MB‐231 cells with an OGT inhibitor OSMI‐1 led to a marked reduction in YAP O‐GlcNAcylation, diminished interaction with EIF3H as determined by both co‐immunoprecipitation and PLA assay, and a decrease in YAP protein levels (Figure 3G–I; Figure S4C, Supporting Information). Moreover, the application of the OGT inhibitor resulted in accelerated YAP protein degradation and increased ubiquitylation, underscoring OGT's crucial role in modulating YAP stability through its interaction with EIF3H (Figure 3J,K). Most notably, the inhibition of OGT in ACM‐treated MDA‐MB‐231 cells significantly curtailed cell proliferation and colony formation, further affirming the essential function of OGT in maintaining YAP stability (Figure 3L,M; Figure S5D,E, Supporting Information). To investigate whether additional regulatory mechanisms, such as the interplay between YAP phosphorylation and ubiquitylation, contribute to YAP ubiquitylation,^[^ ^57^, ^58^ ^]^ we analyzed phosphorylation at S127 in MDA‐MB‐231 cells treated with SCM or ACM. The results indicated that phosphorylation levels at S127 were not significantly different between these conditions (Figure S5F, Supporting Information), suggesting that phosphorylation does not play a major role in ACM‐induced changes in YAP ubiquitylation.

**Figure 4:**
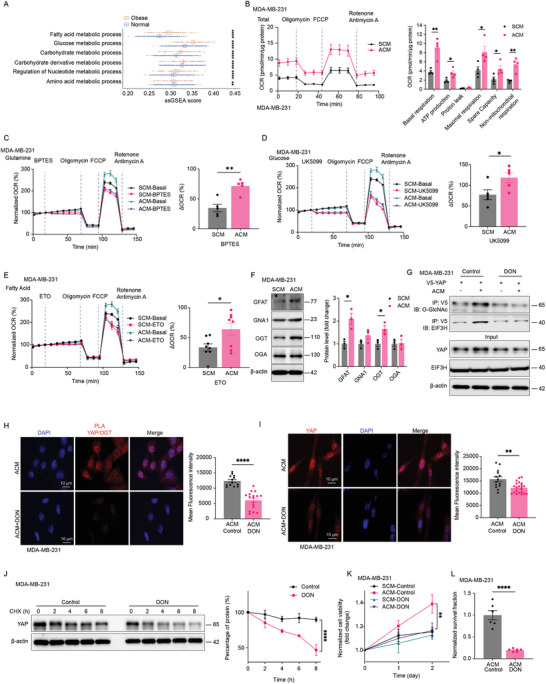
Adipocyte induced metabolic dysregulation leads to enhanced YAP‐O‐GlcNAcylation and subsequently YAP accumulation. A) Comparison of ssGSEA score distributions between obese and normal samples in breast tumors focuses on metabolic processing pathways. P‐values are obtained from Wilcoxon tests. B–E) The Mito stress (B) (*n* = 4) and mitochondrial substrates oxidation (C‐E) were measured for ACM or SCM‐treated MDA‐MB‐231 cells with a Seahorse XF24 Flux Analyzer. Basal respiration, ATP‐linked respiration, maximal and reserve capacities, non‐mitochondrial respiration for Mito stress and maximal respiration change for mitochondrial substrates oxidation were determined ((C‐D) *n* = 5, (E) *n* = 8). F) Hexosamine biosynthesis pathway‐related enzymes, including GFAT, GNA1, OGT, and OGA protein levels, were determined and quantified in ACM and SCM‐treated MDA‐MB‐231 breast cancer cells (*n* = 3). G) ACM‐conditioned MDA‐MB‐231 cells were treated with GFAT inhibitor DON, and both YAP O‐GlcNAcylation and the interaction between YAP and EIF3H were determined by co‐immunoprecipitation. H) ACM‐conditioned MDA‐MB‐231 cells were treated with GFAT inhibitor DON and then incubated with mouse anti‐YAP and rabbit anti‐OGT antibodies. Duolink PLA was then performed and red dots indicate the interaction of the two proteins. The red fluorescence was also quantified (Control *n* = 13, DON *n* = 17). I) ACM‐conditioned MDA‐MB‐231 cells were treated with GFAT inhibitor DON and Immunofluorescence staining of YAP expression (red) was measured and quantified (Control *n* = 14, DON *n* = 22). J) GFAT inhibitor DON treated ACM cultured MDA‐MB‐231 cells were incubated with cycloheximide and YAP protein turnover were determined (*n* = 3). K,L) MDA‐MB‐231 cells were cultured with adipocytes conditioned medium and treated with DON for 24 h, CCK8 based cell proliferation (K) (*n* = 4) and colony formation (L) (*n* = 6) were determined. Data (mean ± SEM) are representative of at least three independent experiments. *p < 0.05, **p < 0.01, ***p < 0.001, and ****p < 0.0001, by multiple unpaired t‐test or two‐way ANOVA followed with Tukey's multiple comparisons test. GFAT, Glutamine fructose‐6‐phosphate amidotransferase. GNA1, glucosamine‐phosphate N‐acetyltransferase 1. OGT, O‐linked N‐acetylglucosamine (GlcNAc) transferase. OGA, O‐GlcNAcase.

In **Figure** **4**A, the metabolic reprogramming pathway is highlighted as a significant altered pathway in obese breast cancer patients. It's established that the enzyme OGT catalyzed O‐GlcNAcylation is a process highly sensitive to cellular nutritional status. This is due to OGT's substrate, UDP‐GlcNAc, which is a product of the hexosamine biosynthetic pathway (HBP) and is directly influenced by the influx of nutrients like glucose, glutamine, acetyl‐CoA, and uridine, meaning changes in nutrient availability can directly affect OGT activity. In this context, we examined mitochondrial stress in ACM‐treated MDA‐MB‐231 cells and observed significant mitochondrial reprogramming, including increased respiration and ATP production (Figure 4B). These findings highlight a distinct bioenergetic profile in cells under obese versus lean conditions. Further investigation into substrate dependency revealed a significant increase in glutamine metabolism in ACM‐treated cells, along with enhanced glucose and fatty acid metabolism, which collectively contribute to elevated glutamine utilization (Figure 4C–E). Importantly, similar results were observed in the 4T1 and HCC1954 cell lines (Figure S6A–H, Supporting Information), further validating these findings across different TNBC models. Concurrently, we observed upregulation of several key genes involved in the HBP, which enhanced OGT expression and functionality, while O‐GlcNAcase (OGA) levels remained unchanged (Figure 4F). To determine the causal relationship, we inhibited the HBP flux in ACM‐cultured MDA‐MB‐231 cells using a Glutamine‐Fructose‐6‐Phosphate Amidotransferase (GFAT) inhibitor, 6‐diazo‐5‐oxo‐norleucine (DON). This treatment resulted in reduced YAP O‐GlcNAcylation (Figure 4G,H), diminished EIF3H interaction (Figure 4G; Figure S6I,J, Supporting Information), decreased YAP accumulation (Figure 4I) and increased YAP turnover (Figure 4J). Additionally, a proliferation and clonogenic formation assay revealed that GFAT inhibition significantly reduced the proliferation rate and colony formation of ACM‐cultured MDA‐MB‐231 cells compared to the SCM group, highlighting the dependency on the HBP pathway for increased YAP O‐GlcNAcylation (Figure 4K,L; Figure S6K,L, Supporting Information).

### 2.4 Molecular Docking Analysis and Experimental Validation of the Interplay between OGT and EIF3H in Regulating YAP Protein Stability

**Figure 5:**
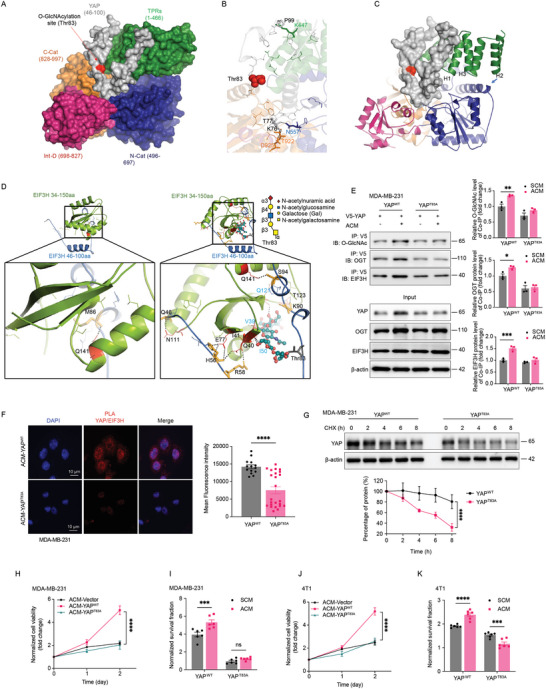
3D Structural modeling and functional studies reveal how glycosylation and ubiquitylation orchestrate adipocyte‐induced YAP accumulation. A–C) Molecular docking simulations elucidate the interaction between the YAP fragment spanning amino acid residues Q46 – E100 and OGT amino acid residues T315 – K1028. (A) a surface model of YAP (Gray) juxtaposed with OGT domains (TPRs in green, N‐Cat in blue, Int‐D in pink, and C‐Cat in orange), (B) An enlarged depiction highlighting the predicted interacting residues. (C) Display of YAP in ribbon rendering, with OGT represented as a surface, offering comprehensive insights into their molecular interaction. D) The structural model, derived from molecular docking simulations, elucidates the intermolecular interactions between the YAP (Q46‐E100) TEAD domain and EIF3H (S34‐L150) amino acid residues, particularly within the JAB/MP domain. The left enlarged region reveals the predicted interacting residues between YAP and EIF3H in the absence of glycosylation on YAP, while the right panel demonstrates an augmented interaction between YAP and EIF3H upon the addition of a glycan moiety on Thr83 of YAP. Notably, the presence of Galactose and N‐acetylmuramic acid on Thr83 facilitates the formation of three hydrogen bonds with EIF3H, as depicted. E) The interaction between YAP with EIF3H and OGT by coimmunoprecipitation of ectopic V5‐YAP^WT^ and V5‐tagged YAP^T83A^ mutant in ACM‐treated MDA‐MB‐231 cells was determined. The O‐GlcNAcylation status of V5‐YAP^WT^ and V5‐tagged YAP^T83A^ mutant were also determined (*n* = 3). F) ACM‐conditioned MDA‐MB‐231‐YAP^WT^ and MDA‐MB‐231‐YAP^T83A^ cells were incubated with mouse anti‐YAP and rabbit anti‐EIF3H antibodies. Duolink PLA was then performed and red dots indicate the interaction of the two proteins. The red fluorescence was also quantified (YAP^WT^ *n* = 14, YAP^T83A^ *n* = 24). G) MDA‐MB‐231‐YAP^WT^ and MDA‐MB‐231‐YAP^T83A^ cells were incubated with cycloheximide, and YAP protein turnover was determined (*n* = 3). H–K) MDA‐MB‐231 cells (H‐I) and 4T1 cells (J‐K) with stably expressing V5‐YAP^WT^ and V5‐tagged YAP^T83A^ mutant were cultured using adipocytes conditioned medium or stromal‐cell conditioned medium, CCK8‐based cell proliferation (H and J) (*n* = 4), and colony formation (I and K) (*n* = 6) was determined. Data (mean ± SEM) are representative of at least three independent experiments. *p < 0.05, **p < 0.01, ***p < 0.001, and ****p < 0.0001, by multiple unpaired T‐test or two‐way ANOVA followed with Tukey's multiple comparisons test.

Our recent research has elucidated the role of EIF3H as a deubiquitinase that mitigates proteasomal degradation of YAP.^[^ ^55^ ^]^ To comprehensively address the mechanism by which the interplay of OGT‐mediated YAP‐ GlcNAcylation and EIF3H‐mediated deubiquitylation regulates YAP stability control, we conducted structural modeling of the interaction between YAP, OGT, and EIF3H by integrated use of docking simulations followed by experimental validation. The models in **Figure** **5**A–C illustrate the interactions between the amino acids of OGT and the YAP domain. Our docking simulations identified potential binding sites and energy dynamics of the YAP‐OGT interaction, with the lowest energy and ligand RMSD at ‐217.94 kJ mol^−1^ and 64.86 Å, respectively. Key residues such as K76, T77, and P99 of YAP formed covalent interactions with K447, N557, T922, and D925 of OGT. This interface was further stabilized by additional hydrophobic interactions involving residues Q53, H56, M73, N74, P75, Q82, P85, M86, F95, P98, of YAP, and Q402, C407, N434, E437, T444, H558, K634, F868, A870, V871, V895, A896, and K898 of OGT. Particularly, the interaction near the O‐GlcNAcylation site on Thr83 of YAP was significant. Further, we conducted an in‐depth exploration into the molecular interactions between deubiquitinase EIF3H and YAP, elucidating the specific interaction site of YAP with and without O‐GlcNAcylation moiety on Thr83. Initial simulations without the glycan showed a single hydrogen bond between M86 of YAP and Q141 of EIF3H, with a calculated binding energy of ‐254.92 kJ mol^−1^. Upon introducing core 1 of the glycan moiety, our observations indicated stronger interactions, as detailed in Figure 5D (left panel) and Figure S7A (Supporting Information). Addition of more sugars to core 1 increased the interaction, as shown in the enhanced docking results in Figure 5D (right panel). The key residues involved in hydrogen bonding between YAP and EIF3H were Q46, H56, R58, K90, S94, on YAP, and Q40, I41, E77, N111, T123, Q141, on EIF3H. Notably, glycosylated Thr83 of YAP interacted with V39, I50, and Q121 of EIF3H, significantly increasing binding affinity to ‐273.09 kJ mol^−1^ and forming thirteen hydrogen bonds, thereby strengthening the YAP‐EIF3H interaction significantly. This comprehensive analysis suggested the critical role of O‐GlcNAcylated Thr83 in enhancing the interaction and stability of YAP.

To experimentally validate the direct impact of O‐GlcNAcylation at Threonine 83 (T83) of YAP on its association with EIF3H, we developed MDA‐MB‐231 cell lines stably expressing either V5‐YAP^WT^ and or a mutant form V5‐YAP^T83A^. The mutation of polar hydrophobic threonine to a structural similar non‐polar hydrophobic alanine (YAP^T83A^) had markedly lower levels of YAP O‐GlcNAcylation, and diminished interaction with EIF3H (Figure 5E,F), which led to an increased turnover of YAP (Figure 5G). Despite an observed increase in OGT expression in both MDA‐MB‐231 cells expressing wild‐type YAP (MDA‐MB‐231‐YAP^WT^) and the T83A mutant (MDA‐MB‐231‐YAP^T83A^) cultured in adipocyte‐conditioned medium (ACM), the expected YAP accumulation did not occur in the MDA‐MB‐231‐YAP^T83A^ cells (Figure S7B, Supporting Information). Additionally, the presence of YAP^T83A^ significantly inhibited the ACM‐induced increase in tumor proliferation rates (Figure 5H–K; Figure S7C,D, Supporting Information). Previous studies have suggested that S109 and T241 are key sites for O‐GlcNAcylation of YAP.^[^ ^31^, ^32^ ^]^ To evaluate the individual contributions of these sites relative to T83, we generated MDA‐MB‐231 cells stably expressing YAP mutants for S109A, T241A, and T83A. Immunoprecipitation (IP) and blotting experiments revealed that while ACM‐induced YAP O‐GlcNAcylation was partially reduced across all mutant groups, the ACM‐induced interaction between YAP and EIF3H was specifically abolished in the T83A mutant, but not in the S109A or T241A mutants (Figure S7E, Supporting Information). These findings suggest that Thr 83 is uniquely critical for mediating the ACM‐induced YAP‐EIF3H interaction and its associated stabilization. Additionally, the presence of YAP^T83A^ significantly inhibited the ACM‐induced increase in tumor proliferation rates, whereas the S109A and T241A mutations had no significant impact under the same conditions (Figure S7F, Supporting Information). These results underscore the functional importance of Thr 83 in mediating the pro‐tumorigenic effects of ACM‐induced YAP activation.

### 2.5 Pharmacological Dissection Identifies Inhibition of HBP‐OGT‐YAP Axis Rescue the Adipocyte Associated Metabolic Disorder through Suppressing the Interplay between Glycosylation and Ubiquitylation of YAP

**Figure 6:**
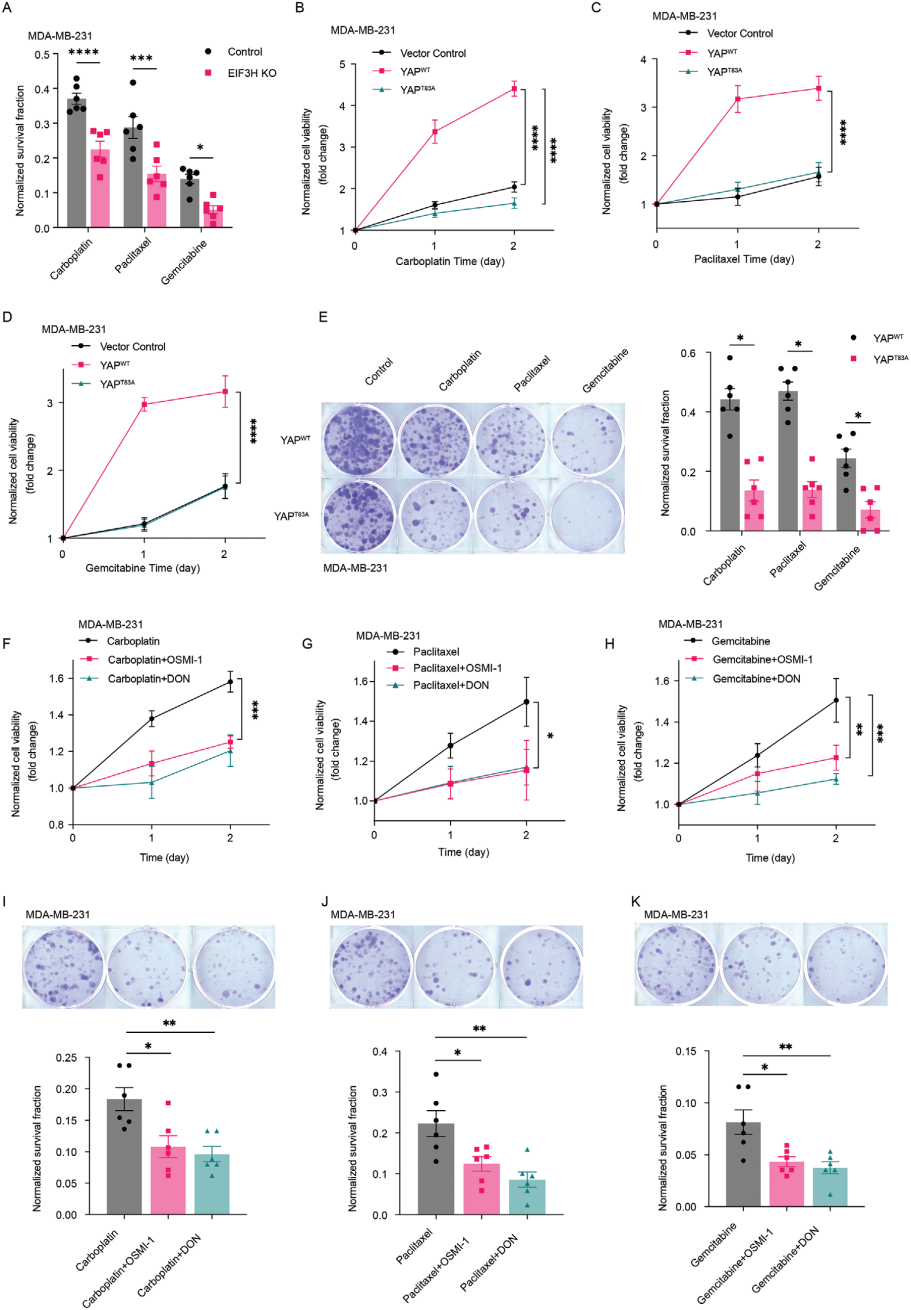
Pharmacological dissection identifies suppressing HBP‐OGT‐YAP axis resensitizes adipocyte associated TNBC cells in response chemotherapeutic agents. A) MDA‐MB‐231‐Control and MDA‐MB‐231‐EIF3H KO cells were cultured with ACM medium and exposed to 25 µg ml^−1^ Carboplatin, 5 µm Gemcitabine, and 500 nm Paclitaxel for 24 h, colony formation was determined (*n* = 6). B–E) MDA‐MB‐231‐Vector control, MDA‐MB‐231‐YAP^WT^, and MDA‐MB‐231‐YAP^T83A^ cells were cultured with ACM medium and exposed to 25 µg ml^−1^ Carboplatin, 5 µm Gemcitabine, and 500 nm Paclitaxel for 24 h, cell proliferation rate (B‐D) (*n* = 4) and clonogenic survival (E) (*n* = 6) was determined and quantified. F–K) CCK8 based cell proliferation rates and colony formation were assessed after treating ACM cultured MDA‐MB‐231 cells with 1 µm OSMI‐1 or DON, and additionally with 25 µg ml^−1^ Carboplatin (F and I), 500 nm Paclitaxel (G and J), or 5 µm Gemcitabine (H and K) for 24 h ((F‐H) *n* = 4, (I‐K) *n* = 6). Data (mean ± SEM) are representative of at least three independent experiments. *p < 0.05, **p < 0.01, ***p < 0.001, and ****p < 0.0001, by multiple unpaired t‐test or two‐way ANOVA followed with Tukey's multiple comparisons test.

To investigate whether obesity‐induced metabolic disorders mediate the interaction between O‐GlcNAcylation and deubiquitylation of YAP, influencing therapy responses in triple‐negative breast cancer, we established stable EIF3H knockdown (shEIF3H) as well as CRISPR/Cas9‐mediated EIF3H knockout (EIF3H KO) in MDA‐MB‐231 and 4T1 cell lines (Figure S8A, Supporting Information). Our findings (**Figure** **6**A; Figure S8B–D, Supporting Information) showed that both EIF3H KO (Figure 6A; Figure S8B, Supporting Information) and shEIF3H (Figure S8C,D, Supporting Information) TNBC cells overcame adipocyte‐conditioned medium (ACM)‐induced pan‐chemotherapy resistance. Furthermore, we observed similar re‐sensitizing effects in MDA‐MB‐231 cells expressing a V5‐tagged YAP^T83A^, a mutant deficient in O‐GlcNAcylation (Figure 6B–E; Figure S8E–I, Supporting Information). Given that O‐GlcNAcylation of YAP directly influences EIF3H recruitment, which in turn affects YAP stability, we explored the potential re‐sensitizing effects of inhibiting O‐GlcNAc transferase (OGT). Remarkably, inhibition of OGT activity using OSMI‐1 led to increased sensitivity of ACM‐treated MDA‐MB‐231 and 4T1 cells to multiple chemotherapy agents, including carboplatin, paclitaxel, and gemcitabine (Figure 6F–K; Figure S8J–O, Supporting Information). These results underscore the critical role of the interplay between glycosylation and ubiquitylation of YAP in modulating the therapeutic response of TNBC. Building on these findings, we employed pharmacological blockade strategies to assess the impact of inhibiting the hexosamine biosynthetic pathway (HBP) influx or glutamine metabolism on chemotherapy resistance. Treatment with GFAT inhibitors DON, by targeting the HBP pathway, we observed that the cells were again re‐sensitized to chemotherapy (Figure 6F–K; Figure S8J–O, Supporting Information). Collectively these data demonstrate that targeting metabolic pathways, specifically through the modulation of O‐GlcNAcylation and glutamine metabolism, presents a viable strategy to overcome chemotherapy resistance in TNBC.

### 2.6 Retatrutide Overcomes Chemotherapy Resistance Due to Obesity in Preclinical TNBC Models

**Figure 7:**
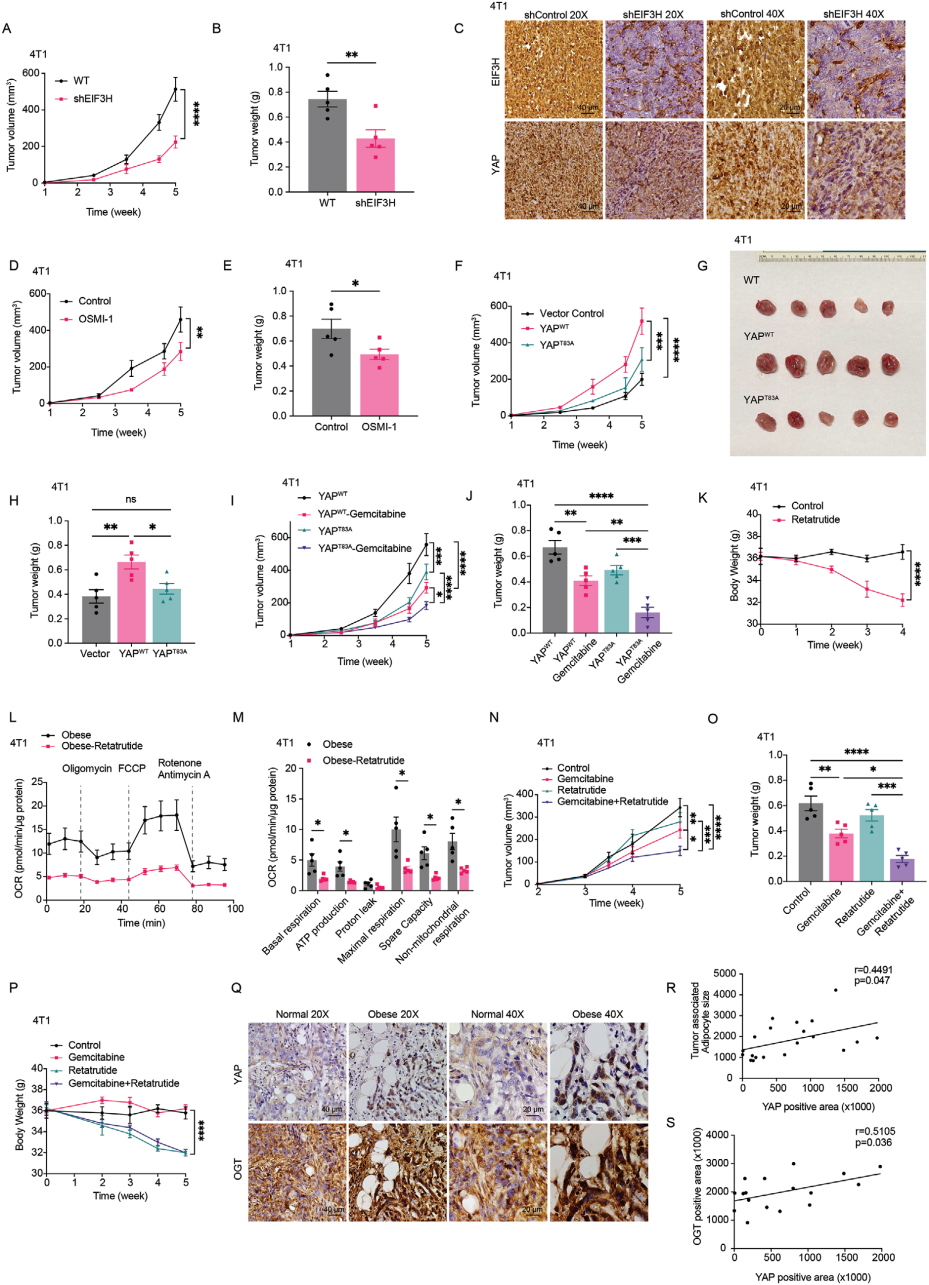
Retatrutide enhances chemosensitivity in obese TNBC models by modulating metabolic barriers to treatment. A,B) 4T1‐shCon and 4T1‐shEIF3H breast cancer cells were orthotopically injected into the right fourth mammary gland of the HFD‐fed BALB/c wild‐type (WT) mice. The tumor growth curve was plotted (A), and tumor weight was measured at the end point (B) (*n* = 5). C) Tumors were fixed in 4% buffered formaldehyde, paraffin‐embedded, and processed for EIF3H and YAP staining. D,E) 4T1 cells were orthotopically injected into the right fourth mammary fat pad in HFD‐fed BALB/c mice and allowed to grow around 100 mm^3^, followed by injection of OSMI‐1 (5 mg kg^−1^, i.p.) two times/week. Phosphate‐buffered saline (PBS) was used in control mouse group. Tumor growth (D) and tumor weight (E) were plotted (*n* = 5). F–H) 4T1 control, 4T1‐YAP^WT^ and 4T1‐YAP^T83A^ tumors were orthotopically injected and harvested 35 days after tumor challenge in HFD fed mice and analyzed. The tumor growth curve was plotted (F), and tumor weight (G‐H) was measured at the endpoint (*n* = 5). I,J) 4T1‐YAP^WT^ and 4T1‐YAP^T83A^ cells were injected into the right fourth mammary fat pad in HFD‐fed BALB/c mice and allowed to grow around 100 mm^3^, followed by injection of gemcitabine (5 mg kg^−1^, i.p.) for two times/week. PBS was used in control groups. Tumor growth (I) was plotted, and tumor weight (J) was measured at the endpoints (*n* = 5). K–M) 4T1 cells were orthotopically injected into the right fourth mammary fat pad in HFD‐fed mice and allowed to grow around 100 mm^3^, followed by injection of Retatrutide (5 mg kg^−1^, i.p.) for two times/week. Mice body weights were monitored (K) and Control or Retatrutide‐treated 4T1 tumors were harvested, digested and assayed for mitochondrial respiration (L‐M) (*n* = 5). N–P) MDA‐MB‐231 cells were orthotopically injected into the right fourth mammary fat pad in HFD fed nude mice and allowed to grow around 100 mm^3^, followed by injection of gemcitabine (5 mg kg^−1^, i.p.) and Retatrutide (5 mg kg^−1^, i.p.) for two times/week. PBS were used in control groups. Tumor growth (N) was plotted, tumor weight was measured (O), and mice body weight was monitored (P) (*n* = 5). Q–S) Representative staining of YAP and OGT in obese and non‐obese human TNBC tissue sections (*n* = 17). The analysis using ImageJ shows a positive correlation between adipocyte size and YAP expression (R) as well as YAP and OGT (S). Data (mean ± SEM) are representative of at least three independent experiments. *p < 0.05, **p < 0.01, ***p < 0.001, and ****p < 0.0001, by multiple unpaired t‐test or one‐way or two‐way ANOVA followed with Tukey's multiple comparisons test.

To investigate the influence on tumor progression through the YAP O‐GlcNAcylation and subsequently EIF3H guided stabilization, we established EIF3H knockdown (shEIF3H) in 4T1 and EIF3H knockout (EIF3H KO) in MDA‐MB‐231, using cells with an empty vector as the control group. Both the control and the EIF3H KD/KO, were injected subcutaneously into the mammary fat pads of HFD‐fed female BALB/c or nude mice. We monitored tumor growth by measuring tumor volumes using the formula V = (W^2 × L)/2, where V is the volume, W the width, and L the length of the tumor. By the 35th day after injection, the mice that received the 4T1‐shEIF3H cells showed significantly reduced tumor growth rates and tumor weights compared to those in the control group (**Figure** **7**A,B). Further analysis through immunohistochemical staining of the tumor tissues indicated that EIF3H suppression reduced YAP accumulation (Figure 7C). To extend these findings, we employed the MDA‐MB‐231 xenograft model in obese mice to validate our results. In obese nude mice, tumors derived from MDA‐MB‐231‐ CRISPR/Cas9‐mediated EIF3H knockout (EIF3H KO) cells also exhibited significantly slower growth and smaller tumor sizes compared to the control group (Figure S9A,B, Supporting Information). To specifically investigate the role of YAP O‐GlcNAcylation in promoting tumor growth in vivo in obese mice, we injected HFD‐fed obese mice with either 4T1 cells (BALB/c mice) (Figure 7D,E) or MDA‐MB‐231 cells (nude mice) (Figure S9C,D, Supporting Information) and treated them with a 5 mg kg^−1^ OGT inhibitor twice weekly. Results showed significant tumor growth suppression in response to OGT inhibition, underscoring the critical role of YAP O‐GlcNAcylation and deubiquitylation in obesity‐driven tumor progression. To investigate the role of the YAP O‐GlcNAcylation pathway in an alternative obesity‐related TNBC model, we conducted experiments using E0771 cells in C57BL/6 mice. E0771 cells were injected into the mammary fat pads of C57BL/6 mice fed a high‐fat diet (HFD). Treatment with the OGT inhibitor OSMI‐1 significantly reduced tumor growth and tumor weight in HFD‐fed mice, consistent with the results observed in the 4T1‐BALB/c model (Figure S9E,F, Supporting Information).To determine if the HBP‐OGT‐EIF3H‐YAP axis plays a role in promoting TNBC metastasis, we utilized a spontaneous lung metastasis model by injecting 4T1 cells into the tail vein of BALB/c mice. Administration of the OGT inhibitor OSMI‐1 significantly reduced the number of lung metastasis nodules. (Figure S9G, Supporting Information). Furthermore, 4T1 cells harboring the YAP T83A mutation, which impairs YAP O‐GlcNAcylation, also exhibited a pronounced inhibitory effect on tumor metastasis (Figure S9H, Supporting Information), suggesting that the HBP‐OGT‐EIF3H‐YAP axis contributes to TNBC metastasis and highlighting the potential of targeting this pathway to suppress metastatic progression. To assess whether targeting YAP O‐GlcNAcylation could enhance the efficacy of current chemotherapies in obese patients, we investigated the anti‐tumor effects of gemcitabine in a high‐fat diet (HFD) BALB/c mouse model (Figure 7F–J). In this model, mice were injected with either 4T1 cells expressing wild‐type YAP (4T1‐YAP^WT^) or cells expressing the YAP mutation T83A (4T1‐YAP^T83A^). Figure 7F–H and Figure S9H (Supporting Information) showed decreased tumor growth (Figure 7F–G), tumor weight (Figure 7H) and lung metastasis (Figure S9H, Supporting Information) in 4T1‐YAP^T83A^ mutant group. According to Figure 7I,J, the 4T1‐YAP^WT^ tumors also exhibited the highest growth rates and were relatively resistant to gemcitabine treatment. In contrast, the 4T1‐YAP^T83A^ tumors displayed slower growth rates, and when treated with gemcitabine, there was a significant reduction in tumor growth. This suggests that YAP O‐GlcNAcylation may play a critical role in the sensitivity of tumor cells to chemotherapy in the context of obesity.

Figure 4 illustrates that obese‐induced metabolic reprogramming, characterized by increased hexosamine biosynthetic pathway (HBP) flux, leads to increased YAP O‐GlcNAcylation and stability. To explore whether metabolic dysregulation in obese mice could be addressed with the weight loss drug Retatrutide, and if it could enhance the efficacy of anticancer drugs, we administered Retatrutide to obese mice. This treatment led to a gradual reduction in body weight, as shown in Figure 7K. Further analyses of tumor tissues, including assessments of mitochondrial respiration and fuel profiling, revealed that Retatrutide‐treated mice exhibited metabolic corrections (Figure 7L,M). This was further confirmed in nude mice bearing MDA‐MB‐231 tumors, as shown in Figure S9I–K (Supporting Information). Importantly, we identified a significant decrease in YAP O‐GlcNAcylation in tumor tissues from Retatrutide‐treated mice (Figure S9L, Supporting Information), suggesting a potential role for Retatrutide in downregulating the YAP/O‐GlcNAcylation axis. This finding supports the hypothesis that Retatrutide exerts tumor‐inhibitory effects, particularly in obesity‐driven tumors where the hexosamine biosynthetic pathway is highly active. In addition, to assess Retatrutide's potential to boost the anti‐tumor effects of chemotherapy, obese BALB/c mice with 4T1 breast tumors received weekly injections of Retatrutide combined with gemcitabine. The results, depicted in Figure 7N–P, demonstrated that the combination effectively overcame gemcitabine resistance in obese 4T1 mice, resulting in significant reductions in tumor growth (Figure 7N) and weight (Figure 7O). Additionally, a consistent reduction in body weight was noted in these mice (Figure 7P). Similar findings were observed in obese nude mice with MDA‐MB‐231 tumors. As shown in Figure S9M,N (Supporting Information), both Retatrutide and the OGT inhibitor OSMI‐1 sensitized tumor cells to gemcitabine‐induced growth inhibition, resulting in marked suppression of tumor progression. To determine the clinical relevance of YAP O‐GlcNAcylation in obese TNBC patients with, we conducted IHC analyses on a collection of breast cancer tissue samples, which included specimens from 6 obese and 6 lean individuals. This analysis further established a positive correlation between the expression levels of OGT and YAP. (Figure 7Q–S; Figure S9O, Supporting Information). These findings collectively underscore the promising strategy of pharmacologically targeting metabolic reprogramming in obese triple‐negative breast cancer (TNBC) to overcome therapeutic resistance and curb tumor growth.

## 3 Discussion

**Figure 8:**
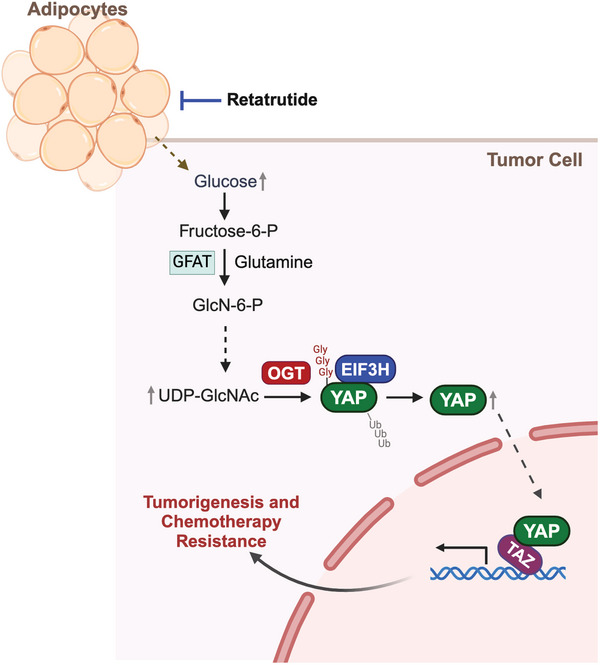
PTM Regulation of YAP in Obesity‐Driven Chemoresistance and the Impact of Retatrutide in TNBC. Increased levels of glucose and glutamine in obese states enhance the activity of GFAT and subsequently the hexosamine biosynthesis pathway (HBP), leading to elevated UDP‐GlcNAc levels. Higher UDP‐GlcNAc levels boost the activity of O‐GlcNAc transferase (OGT), which facilitates the O‐GlcNAcylation of YAP. Enhanced O‐GlcNAcylation leads to reduced ubiquitylation of YAP, by facilitating the interactions with deubiquitinase EIF3H. Consequently, stabilized YAP accumulates and translocates to the nucleus, where it promotes gene transcription that supports tumorigenesis and chemoresistance. The metabolic weight loss drug Retatrutide alters the metabolic environment, reducing the levels of substrates necessary for YAP O‐GlcNAcylation. This reprogramming diminishes YAP's stabilization, thereby enhancing the sensitivity of tumor cells to chemotherapy.

The complex interplay between obesity and the progression of TNBC presents a multifaceted challenge that requires a comprehensive examination of how this relationship impacts both cancer development and therapeutic responses. Within the obese microenvironment, cellular interactions are dynamic and bidirectional, significantly impacting tumor behavior. Our research has highlighted that the dysregulated metabolic pathways prevalent in obesity significantly fuel tumor growth and survival, creating an oncogenic environment that diminishes the efficacy of chemotherapeutic agents (**Figure** **8**). Central to our findings is the role of the transcription factor YAP, whose disruption is crucial in the connection between obesity and TNBC chemoresistance. This study also noted that YAP's altered post‐translational regulation in the context of obesity facilitates a complex network of signaling pathways that promote cancer cell survival and proliferation, thereby complicating treatment outcomes.

### 3.1 YAP Stabilization in the Obese Microenvironment: A Nexus for TNBC Chemoresistance

Obesity is known to impede the effectiveness of chemotherapy, a critical challenge especially in diseases like TNBC.^[^ ^15^ ^]^ The underlying mechanisms involve both direct and indirect alterations in the body's response to cancer treatment. Primarily, obesity changes the pharmacokinetics of drugs by affecting their absorption, distribution, metabolism, and excretion.^[^ ^59^ ^]^ The increased adipose tissue in obese patients can sequester lipophilic drugs, altering their distribution and availability at the tumor site.^[^ ^60^ ^]^ Beyond these pharmacokinetic factors, at the cellular level, recent studies have begun to uncover how obesity contributes to chemotherapy resistance, particularly through the activation of pathways like those involving YAP, recognized as a critical energy sensor, interacts with metabolic processes that are pivotal in cancer metabolism. Research by Dai et al. has demonstrated a global metabolic switch in obesity‐associated tumor cells, highlighting how YAP modulates mitochondrial redox homeostasis.^[^ ^61^ ^]^ Such mechanisms underscore the significance of YAP as a mediator of adipocyte‐driven effects on tumor behavior. Interestingly, increased activation of YAP signaling has been observed in obese settings, but without significant changes in YAP's transcriptional levels, suggesting the presence of alternative regulatory mechanisms at play under these conditions. Our study further explores this phenomenon, finding that an obese microenvironment, characterized by enhanced adipocyte activity, leads to increased YAP stabilization and nuclear activity. Our investigations have demonstrated that in the obese state, cancer‐associated adipocytes drive the enhanced O‐GlcNAcylation of YAP, a process that shields YAP from ubiquitin‐proteasome degradation. This protective mechanism allows YAP to more effectively exert its transcriptional regulatory functions, promoting genes that drive cell proliferation and survival. Consequently, this leads to enhanced resistance to the cytotoxic effects of conventional chemotherapy regimens. This intricate interplay between obesity‐induced metabolic dysregulation and YAP signaling provides a potential target for improving chemotherapy responsiveness in TNBC patients.

### 3.2 Metabolic Dysfunction Induced YAP O‐GlcNAcylation and Aberrant Proteolysis of YAP in Obesity: A Catalyst for TNBC Progression and Chemoresistance

Understanding the mechanism of YAP upregulation in obesity is critical due to its complex regulation by various post‐translational modifications, including phosphorylation, ubiquitylation, SUMOylation, acetylation, and O‐GlcNAcylation.^[^ ^53^, ^54^ ^]^ These modifications intricately control YAP's stability, localization, and transcriptional activity. In this study, we found that obesity‐induced conditions increased YAP stability through enhanced EIF3H deubiquitinase activities. Interestingly, while the general protein synthesis activity, including components of the EIF3 complex, typically increases in nutrient‐rich environments such as those present in obesity, EIF3H protein levels did not show a corresponding increase. However, there was a notable increase in the interaction between EIF3H and YAP, suggesting an alternative regulatory crosstalk mechanism at play.

YAP O‐GlcNAcylation has been established as a regulatory mechanism that modulates YAP phosphorylation, nuclear localization, and transcriptional activation by disrupting its interaction with the upstream kinase LATS1. Previous studies have highlighted key modification sites, such as Thr241 and Ser109, that enhance YAP's pro‐tumorigenic capacity in liver and other cancers.^[^ ^31^, ^32^ ^]^ O‐GlcNAcylation, a modification driven by the hexosamine biosynthetic pathway (HBP), uses UDP‐GlcNAc synthesized from extracellular glucose. Variations in UDP‐GlcNAc and protein O‐GlcNAc levels, which are affected by the availability of glucose, free fatty acids, uridine, and glutamine, establish O‐GlcNAcylation as a vital nutrient sensor and metabolic regulator.^[^ ^62^ ^]^ Notably, Ruan et al. used a proteomic approach to identify a substantial number of potential OGT‐binding proteins, revealing an enrichment of proteins involved in the proteolytic degradation pathway.^[^ ^63^, ^64^ ^]^ This suggests that O‐GlcNAc signaling may directly influence the ubiquitin system. In this study, we demonstrated that in an obese condition, elevated nutrient levels enhance HBP activity in breast tumor cells, thereby increasing the UDP‐GlcNAc pool and the activity of O‐GlcNAc transferase (OGT). This, in turn, leads to increased O‐GlcNAcylation of YAP at threonine 83, which enhances the interaction between EIF3H and YAP. This interaction protects YAP from ubiquitin‐proteasome degradation and facilitates tumorigenesis and chemotherapy resistance. However, this study acknowledges certain limitations: O‐GlcNAcylation may compete with phosphorylation due to structural similarities between phosphate and N‐acetylglucosamine groups. Since phosphorylation of YAP is a signal for its ubiquitylation and subsequent degradation, increased O‐GlcNAcylation in obesity can inhibit this phosphorylation, thereby stabilizing YAP. Alternatively, O‐GlcNAcylation at specific sites on YAP might prevent the attachment of ubiquitin molecules, either through steric hindrance or by inducing conformational changes that shield ubiquitylation sites. Future genetic studies are planned to dissect the distinct impacts of O‐GlcNAcylation on YAP stability by specifically mutating key ubiquitylation/phosphorylation sites on YAP. This will provide a clearer understanding of how O‐GlcNAcylation regulates YAP stability and interacts with EIF3H in breast tumorigenesis and therapeutic outcomes.

In addition, previous findings suggest that EIF3H can interact with and stabilize OGT, enhancing its activity in a deubiquitinase‐dependent manner.^[^ ^65^ ^]^ This mechanism may similarly operate in obesity‐driven TNBC, amplifying OGT activity and YAP O‐GlcNAcylation through the hexosamine biosynthetic pathway. While our study focused on EIF3H‐YAP interactions, future investigations should also explore whether EIF3H‐mediated OGT stabilization contributes to YAP regulation and obesity‐driven tumor progression.

### 3.3 Retatrutide: A Metabolic Drug's Potential in Reversing Chemoresistance in TNBC

In our study utilizing a murine pre‐clinical model, we explored the synergistic potential of combining the metabolic drug Retatrutide with conventional chemotherapeutic agents, specifically gemcitabine and carboplatin, for the treatment of triple‐negative breast cancer. Although recent clinical trials have examined the efficacy of the anti‐diabetic and anti‐obesity medication metformin when combined with chemotherapy in breast cancer patients, these trials found that metformin did not significantly improve chemotherapy efficacy or overall outcomes in patients with metastatic breast cancer without diabetes.^[^ ^66^ ^]^ This discrepancy prompted us to investigate Retatrutide, a drug with a different mechanism of action, to determine if it could offer better results. Administration of Retatrutide led to a gradual reduction in body weight and re‐establishment of metabolic homeostasis, indicating its significant impact on metabolic regulation. Notably, Retatrutide treatment significantly enhanced the sensitivity of TNBC cells to chemotherapy. This heightened chemosensitivity is likely due to Retatrutide‐induced metabolic reprogramming, which alters metabolic pathways often dysregulated in obesity‐associated cancer progression. We speculate that Retatrutide's broader and more profound effects on metabolic regulation and cancer cell metabolism offer a more promising approach for improving chemotherapy efficacy in cancer patients, particularly those with obesity‐related metabolic dysregulation. It is noteworthy that Retatrutide treatment alone exhibited a delayed tumor‐inhibitory effect compared to other therapies, such as Gemcitabine. This could be attributed to the time required for Retatrutide to normalize body weight in obese mice, suggesting that earlier intervention or prolonged treatment might yield more pronounced anti‐tumor effects. Future studies should investigate the long‐term impact of Retatrutide on tumor progression and evaluate its effects in combination with chemotherapies like Gemcitabine to explore potential synergistic benefits.

The observed increase in chemotherapy efficacy in Retatrutide‐treated groups highlights the potential of metabolic drugs to influence key oncogenic pathways, including those regulated by the YAP. As demonstrated, the stabilization of YAP, facilitated through the O‐GlcNAcylation process driven by metabolic changes in obesity, offers a novel target for therapeutic intervention. Retatrutide's dual role in managing weight and modifying biochemical pathways critical to tumor growth and chemoresistance underscores its value. These findings strongly support the inclusion of metabolic modulators such as Retatrutide into combination therapy regimens for TNBC, especially for patients experiencing obesity‐related metabolic dysregulation. However, Retatrutide targets multiple receptors, including glucagon like peptide 1 receptor (GLP1R), gastric inhibitory polypeptide receptor (GIPR), and glucagon receptor (GCGR), which expands its potential mechanisms of action beyond YAP signaling. For example, its effects on systemic glucose and lipid metabolism may indirectly influence the tumor microenvironment by altering nutrient availability, reducing inflammation, or modulating immune responses. These multifaceted metabolic impacts may act in parallel with or independently of YAP inhibition to suppress tumor growth which warrant further studies. In addition, translating these preclinical successes into clinical practice will require comprehensive clinical trials to verify these findings and refine treatment strategies. Such an approach emphasizes the need for a personalized understanding of metabolic and genetic profiles to optimize therapeutic efficacy and enhance clinical outcomes for TNBC patients. This tailored approach could significantly enhance the management of TNBC, offering hope through more effective and customized therapeutic strategies.

## 4 Conclusion

Our study identifies O‐GlcNAcylation at Thr83 as a key modification in obesity‐driven tumor progression, facilitating EIF3H recruitment, YAP stabilization, and tumor‐enhancing functions. This previously uncharacterized interplay between glycosylation and ubiquitylation highlights how obesity‐induced metabolic reprogramming via the hexosamine biosynthetic pathway (HBP) amplifies YAP activity and stability. We also identify Retatrutide, an anti‐obesity agent, as a novel therapeutic intervention that reduces YAP O‐GlcNAcylation and promotes ubiquitylation, disrupting YAP stabilization in the obese tumor microenvironment. These findings provide a clinically relevant framework for targeting obesity‐driven TNBC through metabolic and post‐translational regulation.

## 5 Experimental Section

Sex as a biological variable. This study exclusively examined female mice because the disease modeled is only relevant in females.

### Bioinformatics Analysis

Public cancer patient datasets were analyzed with established bioinformatics methods.^[^ ^67^, ^68^ ^]^ The gene expression matrix of 1092 patients was obtained from the Cancer Genome Atlas (TCGA) and Clinical Proteomic Tumor Analysis Consortium (CPTAC) breast cancer study.^[^ ^69^ ^]^ In the absence of obesity information, the samples were categorized into obese and normal groups based on the expression patterns of 79 obesity‐associated genes. The gene/protein expressions were clustered according to the difference between the average expression of each sample and the average expression of other samples. Gene/protein expression levels corresponding to the listed Hippo proteins were analyzed and heatmaps were generated.

For the single‐sample gene set enrichment analysis (ssGSEA), pathways and gene sets were gathered from the Gene Ontology (GO) annotation. The R package GSVA was utilized to assess the enrichment of each pathway for each sample and computed the ssGSEA score.

### Cell Culture

Human MDA‐MB‐231 and HCC1954 TNBC cell lines, human embryonic kidney HEK293T cell line, mouse 4T1 TNBC cell line, and mouse OP9 preadipocyte cell line were purchased from the American Type Culture Collection (ATCC). MDA‐MB‐231, HEK293T, and 4T1 cells were maintained in Dulbecco's modified Eagle medium (DMEM), and HCC1954 cells were cultured in Roswell Park Memorial Institute 1640 medium supplemented with 10% fetal bovine serum (FBS), 1% of streptomycin (100 U ml^−1^), and penicillin (100 U ml^−1^). OP9 cells were cultured in α‐MEM supplemented with 20% FBS, 1% of streptomycin (100 U ml^−1^), and penicillin (100 U ml^−1^). All cells were cultured at 37 °C in a humidified atmosphere of 5% CO_2_.

### Adipocyte Differentiation and Conditioned Medium Collection

Adipocyte differentiation using murine OP‐9 bone marrow stromal cells was performed as previously reported.^[^ ^70^, ^71^ ^]^ Briefly, confluent cells were trypsinized and plated at 10^4^ cells cm^−2^ in 100 mm dishes. Cells were cultured in DMEM media supplemented with 10% FBS. The following day, the media were replaced with either fresh DMEM supplemented with 10% FBS for stromal cell culture or insulin‐oleate media (IOM; 1.8 mm oleate bound to BSA with a molar ratio of 5.5:1) for adipocyte differentiation. Following adipocyte differentiation, the induction medium was replaced with fresh DMEM supplemented with 10% FBS. Media from bone marrow stromal cells and differentiated adipocytes were harvested after 72 h of culture and subsequently used for experiments.

### Plasmids Information

The wild‐type constructs of EIF3H, shEIF3H (TRCN0000289647 and TRCN0000305068), YAP (#42555), shYAP (#166485, #166486), and LentiCRISPRv2 (#98290) plasmids were purchased from Addgene. For YAP mutagenesis and EIF3H knockout, the corresponding mutations or sgRNAs were introduced with primers by PCR amplification, ligated via Gibson assembly and validated by restriction enzyme digestion and sequencing.

The mutation sequence targeting YAP^T83A^:

T83A‐F: AACGTGCCCCAGgCCGTGCCCATGAGGCTCCG

T83A‐R: ACGGcCTGGGGCACGTTGGCCGTCTTGGGGTT

The mutation sequence targeting YAP^S109A^:

S109A‐F: ACAGGCCgctACTGATGCAGGCACTGCAGGAG

S109A‐R: CATCAGTagcGGCCTGTCGGGAGTGGGATTTG

The mutation sequence targeting YAP^T241A^:

T241A‐F: AAGCCATGgCTCAGGATGGAGAAATTTACTATATAAACC

T241A‐R: ATCCTGAGcCATGGCTTGTTCCCATCCATCAG

The sgRNA sequence targeting human EIF3H:

sghEIF3H‐1F: caccGCGTCCCGCAAGGAAGGTAC

sghEIF3H‐1R: aaacGTACCTTCCTTGCGGGACGC

sghEIF3H‐2F: caccGTATGGCTCATTCGTTACCC

sghEIF3H‐2R: aaacGGGTAACGAATGAGCCATAC

sghEIF3H‐3F: caccGATCAATGTTTACATGGCGA

sghEIF3H‐3R: aaacTCGCCATGTAAACATTGATC

The sgRNA sequence targeting mouse EIF3H:

sgmEIF3H‐1F: caccGGCAGTTGGTAATCTCTAGC

sgmEIF3H‐1R: aaacGCTAGAGATTACCAACTGCC

sgmEIF3H‐2F: caccGGCTCTACCGCCACCTCCTC

sgmEIF3H‐2R: aaacGAGGAGGTGGCGGTAGAGCC

sgmEIF3H‐3F: caccGTTCCCGTTCCCCCAGCACA

sgmEIF3H‐3R: aaacTGTGCTGGGGGAACGGGAAC

### Cell Transfection and Stable Cell Line Construction with Lentivirus Transduction

Cells are transfected with PEI STAR (TOCRIS) Transfection Reagent following the user manual. Generally, cells were seeded the day before transfection to reach 50–80% confluence on the day of transfection. DNA and PEI STAR were mixed in opti‐MEM medium and added to the cells after 10 min‐incubation at room temperature. Cells were collected for analysis 2–3 days after transfection.

Lentiviral particles were generated by co‐transfection of the expression plasmid and lentiviral package plasmids (pVSV‐G, pRRE, and pRSV‐REV) into HEK‐293T with PEI STAR (TOCRIS) following the user manual. Briefly, cells were seeded the day before transfection to reach 50–80% confluence on the day of transfection. DNA and PEI STAR were mixed in opti‐MEM medium and added to the cells after 10 min‐incubation at room temperature. Culture media were harvested 48 h after transfection, filtered through 0.45 µm filters, mixed with polybrene and added to the targeting cell lines for transduction. Two to three days after transduction, the stable cell lines were established by puromycin or blasticidin selection at 2 µg ml^−1^.

### Seahorse Assay

The OCR was measured with a Seahorse XFe24 Flux Analyzer (Agilent, Santa Clara, CA). Cells were seeded in Seahorse plates overnight to reach 90% confluence. For the Mito stress assay, 1 h before the measurement, culture medium was replaced with XF basal medium (Seahorse Bioscience), and the plates were placed into a 37 °C non‐CO_2_ incubator. The OCR was measured over time following injections of 1 µm oligomycin, 1 µm carbonyl cyanide‐4 (trifluoromethoxy) phenylhydrazone (FCCP), and 0.5 µm rotenone/antimycin A (AA). For the substrate activity assay, basal respiration was first established, followed by injection of the relevant pathway inhibitor. Then, the standard XF Cell Mito Stress Test reagents, such as oligomycin, FCCP, and rotenone/antimycin A were injected sequentially. Each assay was focused on a single substrate using the optimized concentration of relevant inhibitor: etomoxir (ETO) (4 µm) to inhibit oxidation of long chain fatty acid (LCFAs), UK5099 (2 µm) to inhibit the oxidation of glucose and/or pyruvate, and BPTES (3 µm) to inhibit oxidation of glutamine. The maximum OCR change was calculated.

### Structure‐Based Molecular Docking

The crystal structure of the OGT was retrieved from the Protein Data Bank with PDB ID 3PE4 (https://www.rcsb.org/structure/3PE4). The 3D structure YAP and EIF3H were created using homology modelling on the SWISS Model web‐server (https://swissmodel.expasy.org/interactive).^[^ ^72^ ^]^ The SWISS‐MODEL structure assessment online server was used for validation (https://swissmodel.expasy.org/assess/).^[^ ^73^ ^]^ Furthermore, following the modeling of YAP structures, their binding patterns were delved into through the utilization of molecular docking methodology. Structural coordinates of YAP and EIF3H fragments covering Q46‐E100 and S34‐L150 were taken from the modeled structures respectively. Covalent glycan‐protein attachment was employed via Charming (https://www.charmm‐gui.org/) to elucidate the pivotal role of glycans in mediating the interaction between YAP and EIF3H, shedding light on their functional significance in molecular recognition and signaling pathways.^[^ ^74^ ^]^ The docking analysis was performed using the HDOCK webserver (http://hdock.phys.hust.edu.cn/), a computational tool recognized for its accuracy in predicting molecular docking interactions. HDOCK utilizes a hybrid approach that combines template‐based modeling and ab initio docking to generate and rank docking models efficiently. The binding energy for each complex was calculated using a scoring function that integrates both knowledge‐based and physics‐based approaches.^[^ ^75^, ^76^, ^77^, ^78^ ^]^ Subsequent to the docking process, meticulous screening was undertaken to discern complexes screened for higher binding affinity. Among these, the most promising docked conformations were singled out for in‐depth analysis. Leveraging advanced molecular visualization tools such as PyMOL and Discovery Studio Visualizer,^[^ ^79^ ^]^ a comprehensive examination was conducted to unravel the intricate network of potential interactions between the ligand and receptor.

### Cell Proliferation Analysis

The cell proliferation analysis was performed using Cell Counting Kit‐8 (CCK8) assay, image‐based cell proliferation analysis, and clone formation assay. For the CCK8 assay, cells were seeded at 1 × 10^3^ cells per well into the 96‐well culture plates. The 10 µl CCK8 solution reagent was added into each well and incubated for 2 h at 37 °C. Then, a microplate reader was used to detect the absorbance at 450 nm. For the colony formation, cells were trypsinized and seeded into six‐well plates at a density of 100–600 cells per well. After fourteen days of incubation, cells were fixed with 4% paraformaldehyde and stained with 0.5% crystal violet. All experiments were conducted three times with three replicates individually.

### Western Blot Analysis

Cells were lysed with 1X Laemmli sample buffer (Bio‐Rad, 1610737), boiled at 95 °C for 5 min, and centrifuged at 14000 × g for 15 min at 4 °C to remove the undissolved cell debris. RIPA extraction reagent combined with protease inhibitors was used to lyse tumor tissues. Protein concentration of cell lysate was measured with the BCA assay kit (ThermoFisher Scientific, 23 225). Twenty micrograms protein was resolved on SDS‐PAGE and transferred to nitrocellulose membrane. Membranes were blocked with PBST containing 5% skim milk for 2 h, incubated with various primary antibodies at 4 °C overnight followed with 5 min‐washing with PBST for 4 times and the corresponding horseradish peroxidase (HRP) conjugated secondary antibody at room temperature for 2 h followed with 5 min‐washing with PBST for 4 times. Chemiluminescence substrate was applied using Clarity Western ECL Substrate (Biorad, 1705061). Blots were imaged using the ChemiDoc Touch Imaging System (Bio‐Rad). Semiquantification of Chemiluminescence was performed using ImageLab (Bio‐Rad). The antibody information was listed below:

YAP: CST #14074; p‐YAP (S127): CST #4911; EIF3H: Santa Cruz sc‐271283; Ubquitin: CST #43124; O‐GlcNAc: Santa Cruz sc‐59623; OGT: Invitrogen PA5‐22071; STT3A: Proteintech 12034‐1‐AP; MGAT1: Sigma SAB1400165; MAN1C1: Sigma SAB1409548; MAN2A2: Sigma HPA077930; GFAT: Santa Cruz sc‐377479; GNA1: Santa Cruz sc‐374519; OGA: sc‐376429.

### Immunoprecipitation

Cells were lysed at 4 °C in ice‐cold cell lysis buffer (25 mm Tris‐HCl, 150 mm NaCl, 1 mm EDTA, 1% NP40, 5% glycerol) with protease inhibitor cocktail (SIGMAFAST, S8830) for 1 h. Cell lysates were cleared by centrifugation at 13000 × g for 30 min. Protein concentration in the supernatant was determined using the BCA Protein assay Kit (Pierce, 23‐225). For immunoprecipitation, cell lysate was incubated with anti‐V5 agarose affinity gel (Sigma) or anti‐YAP agarose affinity gel (Sigma, A7470) overnight at 4 °C on a rotator and washed with PBS for 5 times. The complex was eluted with 100 µg mL^−1^ 3X V5 peptides in PBS for 30 min or boiling in 1X Laemmli sample buffer for 5 min.

### Ubiquitylation Assay

ACM or SCM treated cells were lysed at 4 °C in ice‐cold lysis buffer containing 25 mm Tris‐HCl, 150 mm NaCl, 1 mm EDTA, 1% NP40, and 5% glycerol, supplemented with a protease inhibitor cocktail (SIGMAFAST), for 1 h. The lysates were cleared by centrifugation at 13000 × g for 30 min at 4 °C. Protein concentration in the supernatant was measured using the BCA Protein Assay Kit (Pierce). Cell lysates were incubated with an anti‐YAP antibody (CST, 14074S) overnight at 4 °C on a rotator. Immunocomplexes were washed five times with ice‐cold PBS to remove non‐specific binding. The bound complexes were denatured by boiling in 1X Laemmli sample buffer for 5 min and then subjected to immunoblotting with an anti‐ubiquitin antibody (CST, 43124S) to assess YAP ubiquitylation levels.

### Immunofluorescence Assay

Cells grown on coverslip were fixed with 4% paraformaldehyde in PBS for 20 min and permeabilized by 0.1% Triton X‐100 for 15 min at room temperature. The indicated antibodies diluted in PBST with 0.2% BSA with the dilution factor recommended by the manufacture were incubated with cells overnight at 4 °C. After being washed with PBST 10 min X 3, cells were incubated with Alexa 488 anti‐rabbit /594‐anti‐mouse IgG 1:500 diluted in PBST with 0.2% BSA for 1 h. Cells were washed, stained with 1 ng mL^−1^ DAPI in PBS, washed, mounted, and imaged with BioTek LionHeart fluorescence microscope.

### Immunohistochemistry

The human breast cancer tissue array BC081116f and BR1191 were purchased from TissueArray.Com LLC. Mouse tumor tissues were isolated, embedded, and sectioned. IHC staining of Ki67 (Cell Signaling Technology), YAP (Santa Cruz), and OGT (Thermofisher) using IHC kit (ABCAM) following the user manual. Briefly, slides were deparaffinized, re‐hydrolyzed, implemented antigen retrieval in retrieval buffer (SIGMA) followed by staining procedures. Briefly, the slides were blocked, incubated with primary antibodies, and incubated with the secondary HRP‐ conjugated antibodies and detected with DAB detection kit (ABCAM).

### Duolink Proximity Ligation Assay

The colocalization of YAP with EIF3H and OGT was analyzed by the proximity ligation assay by using Duolink in Situ PLA Kit (Sigma‐Aldrich) and following the manufacturer's protocol. In brief, cells were plated on glass coverslips and fixed with 4% formaldehyde and permeabilized with 0.1% Triton X‐100. Cells were then washed with PBS, incubated with blocking buffer followed by overnight primary antibody incubation at 4 °C. Cells were then incubated with PLA probes, ligation mix, amplification mix and washed twice. The coverslips were mounted using the Duolink in Situ Mounting Medium followed by imaging the cells with BioTek LionHeart fluorescence microscope.

### Protein Stability Assays

To measure the half‐life of YAP, MDA‐MB‐231, and 4T1 cells were seeded in 6‐well plates and cultured with SCM or ACM. After 24 h, cells were treated with 50 µg ml^−1^ protein synthesis inhibitor cycloheximide or 10 µm MG132 (Sigma‐Aldrich) for indicated time points. Western blot was performed to detect protein levels.

### Real‐Time PCR Analysis

Total RNA was extracted using TRIzol reagent (Invitrogen) according to the manufacturer's instructions. The complementary DNA synthesis was performed using SuperScript One‐Step RT‐PCR (Invitrogen). Real‐Time Quantitative Reverse Transcription PCR was used to quantify genes by SYBR Green (Bio‐Rad), and relative abundance of each mRNA was normalized to beta‐actin mRNA. All qPCR reactions were performed in triplicate with the following primers:

Ankrd1 Forward: CACTTCTAGCCCACCCTGTGA

Ankrd1 Reverse: CCACAGGTTCCGTAATGATTT

β‐actin Forward: ATCACCATTGGCAATGAGCG

β‐actin Reverse: TTGAAGGTAGTTTCGTGGAT

Ctgf Forward: ACCGACTGGAAGACACGTTTG

Ctgf Reverse: CCAGGTCAGCTTCGCAAGG

Cyr61 Forward: GGTCAAAGTTACCGGGCAGT

Cyr61 Reverse: GGAGGCATCGAATCCCAGC

Eif3h Forward: TCCTGATTTGTACTGTGTTCGC

Eif3h Reverse: AAGCTACTGCAAAGTTCGGTT

Dlc1 Forward: GGACACCATGATCCTAACACAAA

Dlc1 Reverse: AGCGCAATATCAACAGGGAAC

### Animal Study and Primary Cells Isolation

All animal studies were performed based on guidelines and approval of Emory University Institutional Animal Care and Use Committee (approved IACUC protocol number PROTO202200164). Female 6‐week‐old BALB/c, C57BL/6 or Nude mice purchased from The Jackson Laboratory were fed diets (Research Diets, Inc.) containing 10 kcal% fat/no sucrose low‐fat diet (lean mice) or 60 kcal% fat high‐fat diet (obese mice) for 10 weeks. Mice's body weights were monitored twice a week. Then wild type mouse TNBC 4T1/E0771 cells or human TNBC MDA‐MB‐231 cells (American type culture collection) or engineered shControl/shEIF3H or CRISPR Control/EIF3H KO stable expression breast cancer cells (10^6^) were orthotopically injected into the mammary fat pad, and diets were continued. Tumor volumes were calculated according to the equation: (length) x (width)^2^/2. 4–5 weeks after tumor injection, the mice were sacrificed, and their tumor tissues were harvested for RNA, protein, or histological examination. Lung metastasis model was generated by tail vein injection of 1 × 10^6^ 4T1 wild type or engineered YAP^T83A^ mutant cells. Lung metastases were allowed to develop for up to 4 weeks. Lung tissues were then collected, and the visible metastasis nodules were counted.

To obtain primary tumor cells, the primary tumor tissues were isolated, cut into small pieces and disassociated with 1 mg ml^−1^ Collagenase I (Worthington) and filtered with 100 µm mesh filter. Suspensions were treated with a red blood cell (RBC) lysis buffer to remove RBCs. Isolated cells were seeded in seahorse cell culture plates and incubated at 37 °C for further experiments.

### Statistical Analyses

Data were presented as the mean ± standard error and experiments were performed at least twice. Student t‐test or one‐ or two‐way ANOVA, followed by Tukey multiple comparisons test was applied for statistical tests. Pearson correlation or Spearman correlation was applied for the correlation analysis. Statistical significance was indicated as follows: * *p* < 0.05; ** *p* < 0.01; *** *p* < 0.001, **** *p* < 0.0001. Statistical analyses were carried out with GraphPad Prism 10.0 software.

## Conflict of Interest

The authors declare no conflict of interest.

## Author Contributions

X.C. and Y.Z. contributed equally to this work. X.C. and Y.Z. performed formal analysis, investigation, methodology, wrote the original draft, and edited the draft. L.Z. performed construction for plasmids, Immune‐staining, and colocalization. M.Z. performed bioinformatics. A.U. performed structural modeling and docking simulation analyses. T.G., L.M., and M.T. performed clinical data analysis and editing. S.Z. performed bioinformatics. Y.W. performed conceptualization, drug discovery, data curation, supervision, funding acquisition, project administration, wrote, reviewed, and edited the manuscript.

## Supporting information



Supporting Information

## Data Availability

The data that support the findings of this study are available from the corresponding author upon reasonable request.
