# Supplementary material for: Pharmacological Dissection Identifies Retatrutide Overcomes the Therapeutic Barrier of Obese TNBC Treatments through Suppressing the Interplay between Glycosylation and Ubiquitylation of YAP
Source: Adv Sci (Weinh). 2025 Jan 27;12(11):2407494. doi: 10.1002/advs.202407494 (PMC11923992; doi:10.1002/advs.202407494)
Supplement: Supplementary file 1 — Supporting Information [file ADVS-12-2407494-s001.pdf]

ADVANCED SCIENCE
Open Access

Supporting Information

for *Adv. Sci.*, DOI 10.1002/advs.202407494

Pharmacological Dissection Identifies Retatrutide Overcomes the Therapeutic Barrier of Obese TNBC Treatments through Suppressing the Interplay between Glycosylation and Ubiquitylation of YAP

*Xin Cui, Yueming Zhu, Lidan Zeng, Mengyuan Zhang, Amad Uddin, Theresa W. Gillespie, Lauren E. McCullough, Shaying Zhao, Mylin A. Torres and Yong Wan**

Pharmacological dissection identifies Retatrutide overcomes the therapeutic barrier of obese TNBC treatments through suppressing the interplay between glycosylation and ubiquitylation of YAP

Xin Cui[1†], Yueming Zhu[1,2†], Lidan Zeng[1], Mengyuan Zhang[3], Amad Uddin[1], Theresa W. Gillespie[2,4,5], Lauren E. McCullough[2,6], Shaying Zhao[3], Mylin A. Torres[2,7], and Yong Wan[1,2,5]*

Supplementary Figures 1-9

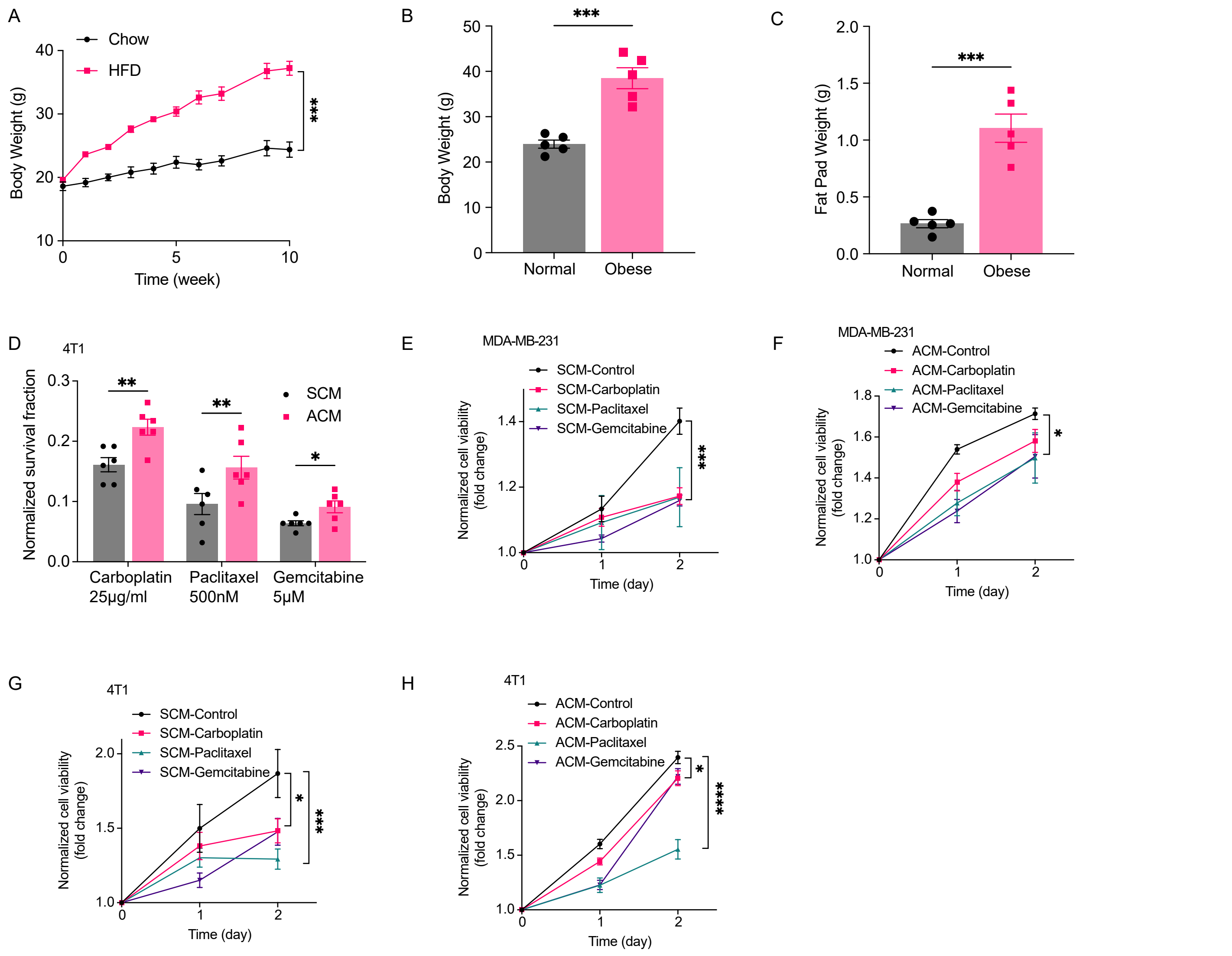

A
Chow
HFD
Body Weight (g)
Time (week)
***
B
Body Weight (g)
Normal
Obese
***
C
Fat Pad Weight (g)
Normal
Obese
***
D
4T1
Normalized survival fraction
SCM
ACM
Carboplatin 25μg/ml
Paclitaxel 500nM
Gemcitabine 5μM
E
MDA-MB-231
SCM-Control
SCM-Carboplatin
SCM-Paclitaxel
SCM-Gemcitabine
Normalized cell viability (fold change)
Time (day)
F
MDA-MB-231
ACM-Control
ACM-Carboplatin
ACM-Paclitaxel
ACM-Gemcitabine
Normalized cell viability (fold change)
Time (day)
G
4T1
SCM-Control
SCM-Carboplatin
SCM-Paclitaxel
SCM-Gemcitabine
Normalized cell viability (fold change)
Time (day)
H
4T1
ACM-Control
ACM-Carboplatin
ACM-Paclitaxel
ACM-Gemcitabine
Normalized cell viability (fold change)
Time (day)

Figure S1

Figure S1. Cancer-associated adipocytes enhance breast cancer progression and therapeutic resistance.

(A-C) Eight-week-old Female BALB/c mice were fed a normal chow diet or high-fat diet (HFD) for 10 weeks. Body weight curve (A) was plotted, body weight (B) and fat pad weight (C) were measured at end point. ($n = 5$). **(D)** Clonogenic cell survival experiments for ACM/SCM treated 4T1 cells with or without exposure to 25 μg/ml Carboplatin, 500 nM Paclitaxel, and 5 μM Gemcitabine, for 24h, as measured by cytotoxicity ($n=6$). **(E-H)** ACM/SCM treated MDA-MB-231 (E-F) and 4T1 cells (G-H) with or without exposure to 25 μg/ml Carboplatin, 500 nM Paclitaxel, and 5 μM Gemcitabine, cell proliferation was measured by CCK8 assay ($n=4$). Data (mean ± SEM) are representative of at least three independent experiments. *$p < 0.05$, **$p < 0.01$, ***$p < 0.001$, and ****$p<0.0001$, by multiple unpaired t-test or two-way ANOVA followed with Tukey's multiple comparisons test.

A

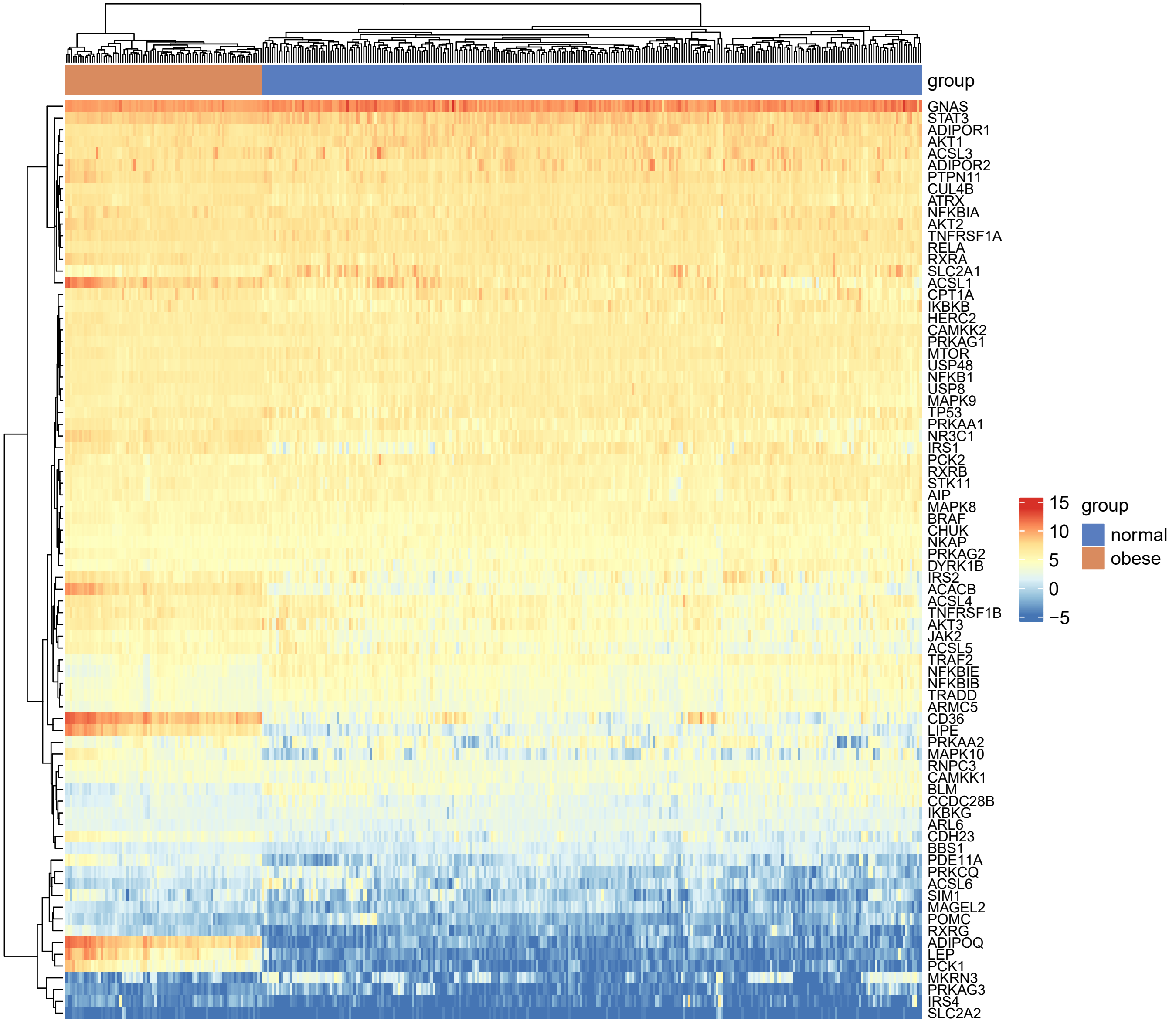
group
GNAS
STAT3
ADIPOR1
AKT1
ACSL3
ADIPOR2
PTPN11
CUL4B
ATRX
NFKBIA
AKT2
TNFRSF1A
RELA
RXRA
SLC2A1
ACSL1
CPT1A
IKBKB
HERC2
CAMKK2
PRKAG1
MTOR
USP48
NFKB1
USP8
MAPK9
TP53
PRKAA1
NR3C1
IRS1
PCK2
RXRB
STK11
AIP
MAPK8
BRAF
CHUK
NKAP
PRKAG2
DYRK1B
IRS2
ACACB
ACSL4
TNFRSF1B
AKT3
JAK2
ACSL5
TRAF2
NFKBIE
NFKBIB
TRADD
ARMC5
CD36
LIPE
PRKAA2
MAPK10
RNPC3
CAMKK1
BLM
CCDC28B
IKBKG
ARL6
CDH23
BBS1
PDE11A
PRKCQ
ACSL6
SIM1
MAGEL2
POMC
RXRG
ADIPOQ
LEP
PCK1
MKRN3
PRKAG3
IRS4
SLC2A2
15
10
5
0
−5
group
normal
obese

B

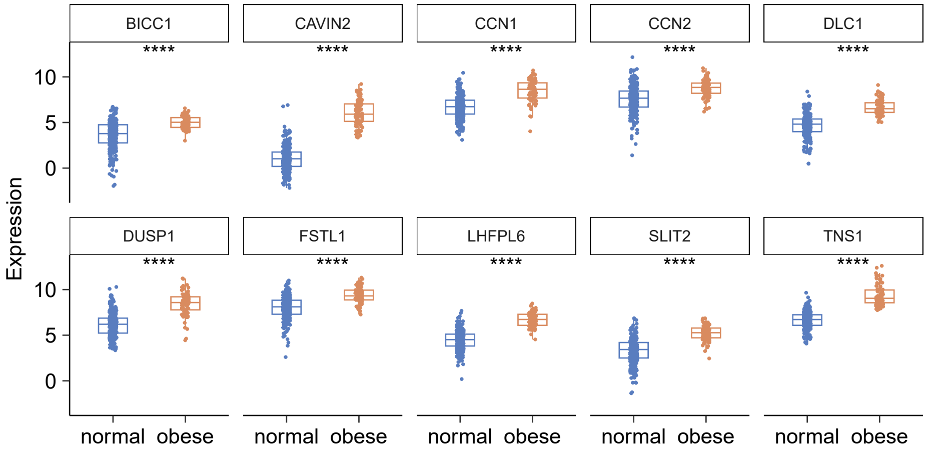
BICC1
CAVIN2
CCN1
CCN2
DLC1
DUSP1
FSTL1
LHFPL6
SLIT2
TNS1
****
Expression
10
5
0
normal
obese

Figure S2

Figure S2. Expression patterns of obesity-associated genes in human breast tumors across obesity and normal weight groups.

(A) Heatmap of the row scaled expression of 79 obesity associated genes of 366 patients from TCGA BRCA cohort. Based on the hierarchical clustering, we classified patients into 282 normal patients and 84 obese patients. **(B)** Box plot displaying the distribution of the scaled expression of key downstream genes in the Hippo-YAP pathway between the normal patients and obese patients. All P-values are from Wilcoxon tests. *: $p < 0.05$; **: $p < 0.01$; ***: $p < 0.001$; ****: $p < 0.0001$.

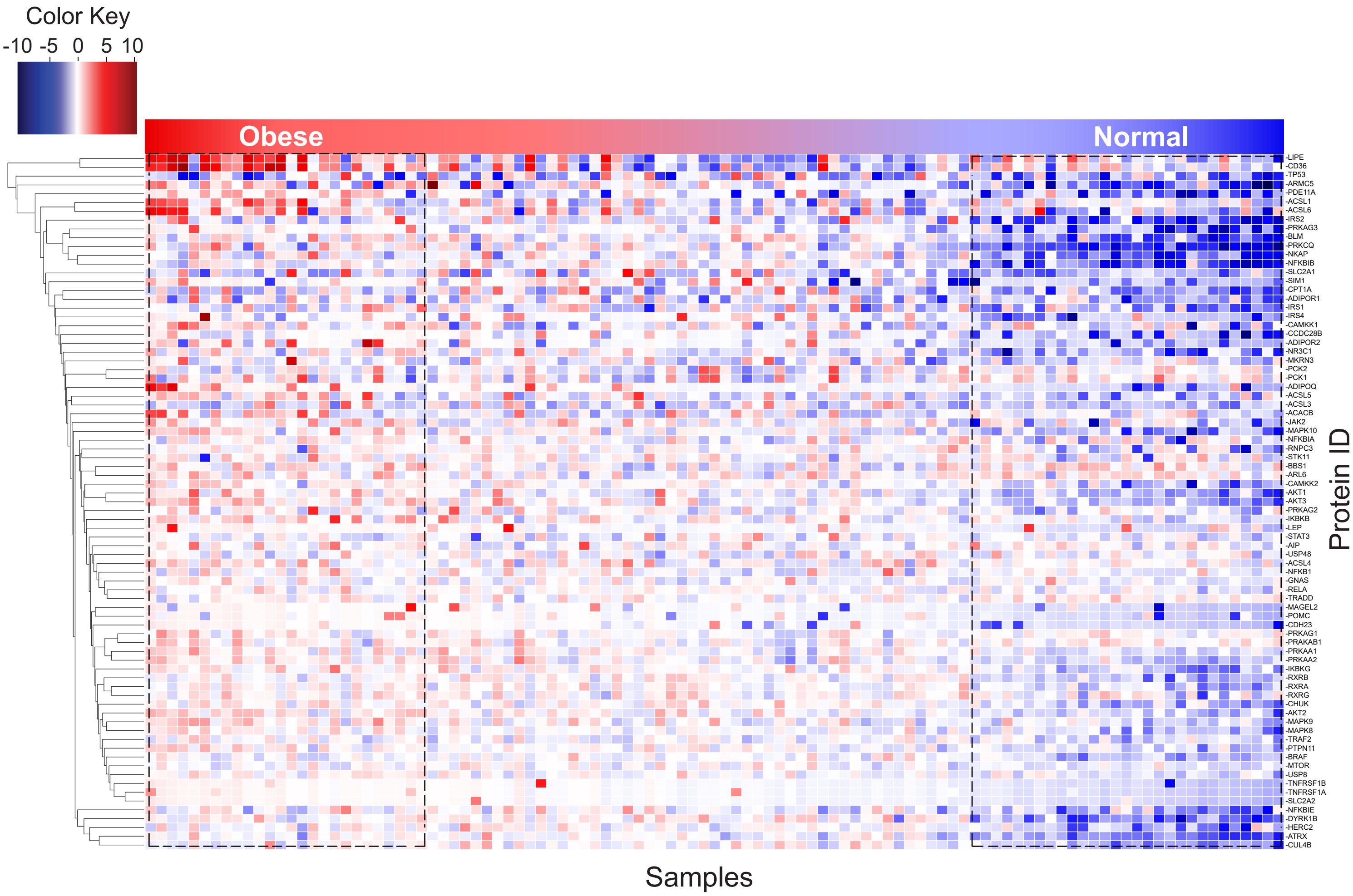
Color Key
-10 -5 0 5 10
Obese
Normal
-LIPE
-CD36
-TP53
-ARMC5
-PDE11A
-ACSL1
-ACSL6
-IRS2
-PRKAG3
-BLM
-PRKCQ
-NKAP
-NFKBIB
-SLC2A1
-SIM1
-CPT1A
-ADIPOR1
-IRS1
-IRS4
-CAMKK1
-CCDC28B
-ADIPOR2
-NR3C1
-MKRN3
-PCK2
-PCK1
-ADIPOQ
-ACSL5
-ACSL3
-ACACB
-JAK2
-MAPK10
-NFKBIA
-RNPC3
-STK11
-BBS1
-ARL6
-CAMKK2
-AKT1
-AKT3
-PRKAG2
-IKBKB
-LEP
-STAT3
-AIP
-USP48
-ACSL4
-NFKB1
-GNAS
-RELA
-TRADD
-MAGEL2
-POMC
-CDH23
-PRKAG1
-PRAKAB1
-PRKAA1
-PRKAA2
-IKBKG
-RXRB
-RXRA
-RXRG
-CHUK
-AKT2
-MAPK9
-MAPK8
-TRAF2
-PTPN11
-BRAF
-MTOR
-USP8
-TNFRSF1B
-TNFRSF1A
-SLC2A2
-NFKBIE
-DYRK1B
-HERC2
-ATRX
-CUL4B
Protein ID
Samples

Figure S3

Figure S3. Obesity-associated protein expression level in human breast tumors across obesity and normal weight groups.

Heatmap and hierarchical clustering display the row-scaled log2 protein expression values of 79 obesity-associated genes from 105 human breast tumors (columns), arranged from left to right by expression level from high (obese) to low (normal). The log2 relative gene expression scale is depicted on the top left.

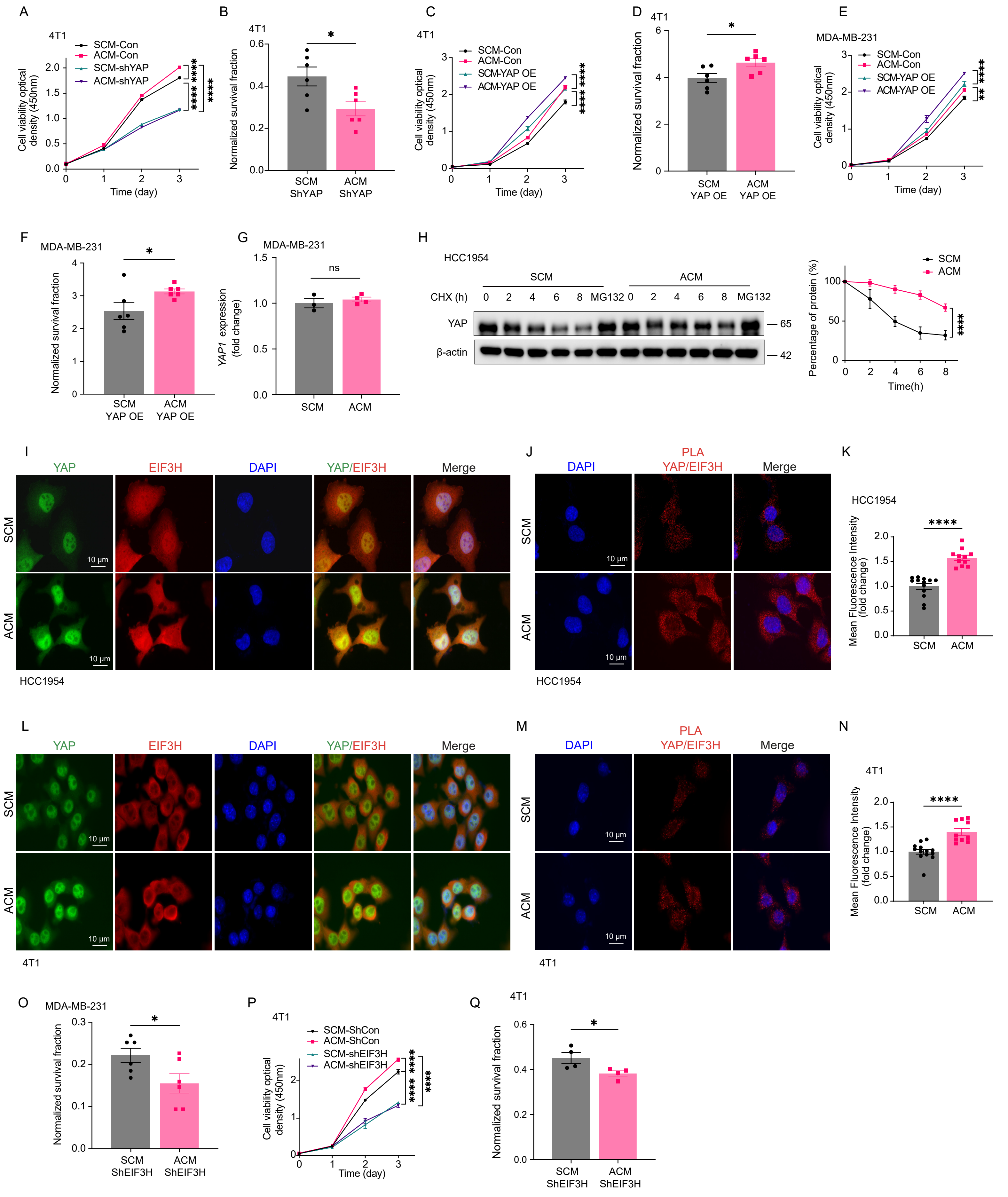

A
4T1
SCM-Con
ACM-Con
SCM-shYAP
ACM-shYAP
Cell viability optical density (450nm)
Time (day)
****
B
4T1
Normalized survival fraction
SCM ShYAP
ACM ShYAP
*
C
4T1
SCM-Con
ACM-Con
SCM-YAP OE
ACM-YAP OE
Cell viability optical density (450nm)
Time (day)
D
4T1
Normalized survival fraction
SCM YAP OE
ACM YAP OE
E
MDA-MB-231
SCM-Con
ACM-Con
SCM-YAP OE
ACM-YAP OE
Cell viability optical density (450nm)
Time (day)
F
MDA-MB-231
Normalized survival fraction
SCM YAP OE
ACM YAP OE
G
MDA-MB-231
YAP1 expression (fold change)
ns
SCM
ACM
H
HCC1954
SCM
ACM
CHX (h) 0 2 4 6 8 MG132 0 2 4 6 8 MG132
YAP
β-actin
— 65
— 42
Percentage of protein (%)
Time(h)
I
YAP
EIF3H
DAPI
YAP/EIF3H
Merge
SCM
ACM
10 μm
HCC1954
J
DAPI
PLA YAP/EIF3H
Merge
HCC1954
K
HCC1954
Mean Fluorescence Intensity (fold change)
L
4T1
M
4T1
N
4T1
Mean Fluorescence Intensity (fold change)
O
MDA-MB-231
Normalized survival fraction
SCM ShEIF3H
ACM ShEIF3H
P
4T1
SCM-ShCon
ACM-ShCon
SCM-shEIF3H
ACM-shEIF3H
Cell viability optical density (450nm)
Time (day)
Q
4T1
Normalized survival fraction
SCM ShEIF3H
ACM ShEIF3H

Figure S4

Figure S4. EIF3H-mediated YAP stabilization modulates cell proliferation and survival in adipocyte-conditioned TNBC cells.

(A-B) 4T1-shCon and 4T1-shYAP cells were cultured with adipocytes conditioned medium or stromal-cell conditioned medium; cell proliferation and survival were measured by CCK8 (A) (n=4) and clonogenic survival assay (B) (n=6). **(C-D)** 4T1-vector control and 4T1-YAP OE cells were cultured using adipocytes conditioned medium or stromal-cell conditioned medium, cell proliferation and survival were measured by CCK8 (C) (n=4) and clonogenic survival assay (D) (n=6). **(E-F)** MDA-MB-231-vector control and MDA-MB-231-YAP OE cells were cultured using adipocytes conditioned medium or stromal-cell conditioned medium, cell proliferation and survival were measured by CCK8 (E) (n=4) and clonogenic survival assay (F) (n=6). **(G)** YAP mRNA levels were determined and quantified in SCM and ACM-treated MDA-MB-231 cells (SCM n=3, ACM n=4). **(H)** HCC1954 cells were treated with cycloheximide or MG132, and YAP protein levels were determined and quantified (n=3). **(I)** Colocalization between YAP and EIF3H and DAPI in HCC1954 cells was measured by immunofluorescence staining, showing a principal overlap of YAP (green) and EIF3H (red) in both nuclei and cytosol. **(J-K)** Slides of SCM or ACM treated HCC1954 cells were incubated with mouse anti-YAP and rabbit anti-EIF3H antibodies. Duolink PLA was then performed and red dots indicate the interaction of the two proteins (J). The fluorescence intensities were quantified in (K) (SCM n=13, ACM n=11). **(L)** Colocalization between YAP and EIF3H and DAPI in 4T1 cells showed a principal overlap of YAP (green) and EIF3H (red) by immunofluorescence staining. **(M-N)** Duolink PLA was performed in SCM or ACM treated 4T1 cells to indicate the interaction of the YAP and EIF3H proteins (M). The fluorescence intensities were quantified in (N) (SCM n=13, ACM n=11). **(O)** MDA-MB-231-shCon and MDA-MB-231-shEIF3H cells were cultured using adipocytes conditioned medium or stromal-cell conditioned medium; clonogenic survival were determined (n=6). **(P-Q)** 4T1-shCon and 4T1-shEIF3H cells were cultured using adipocytes conditioned medium or stromal-cell conditioned medium; cell proliferation and survival were measured by CCK8 (P) and clonogenic survival assay

(Q) (n=4). Data (mean ± SEM) are representative of at least three independent experiments. $^*p < 0.05$, $^{**}p < 0.01$, $^{***}p < 0.001$, and $^{****}p<0.0001$, by unpaired t-test or two-way ANOVA followed with Tukey's multiple comparisons test.

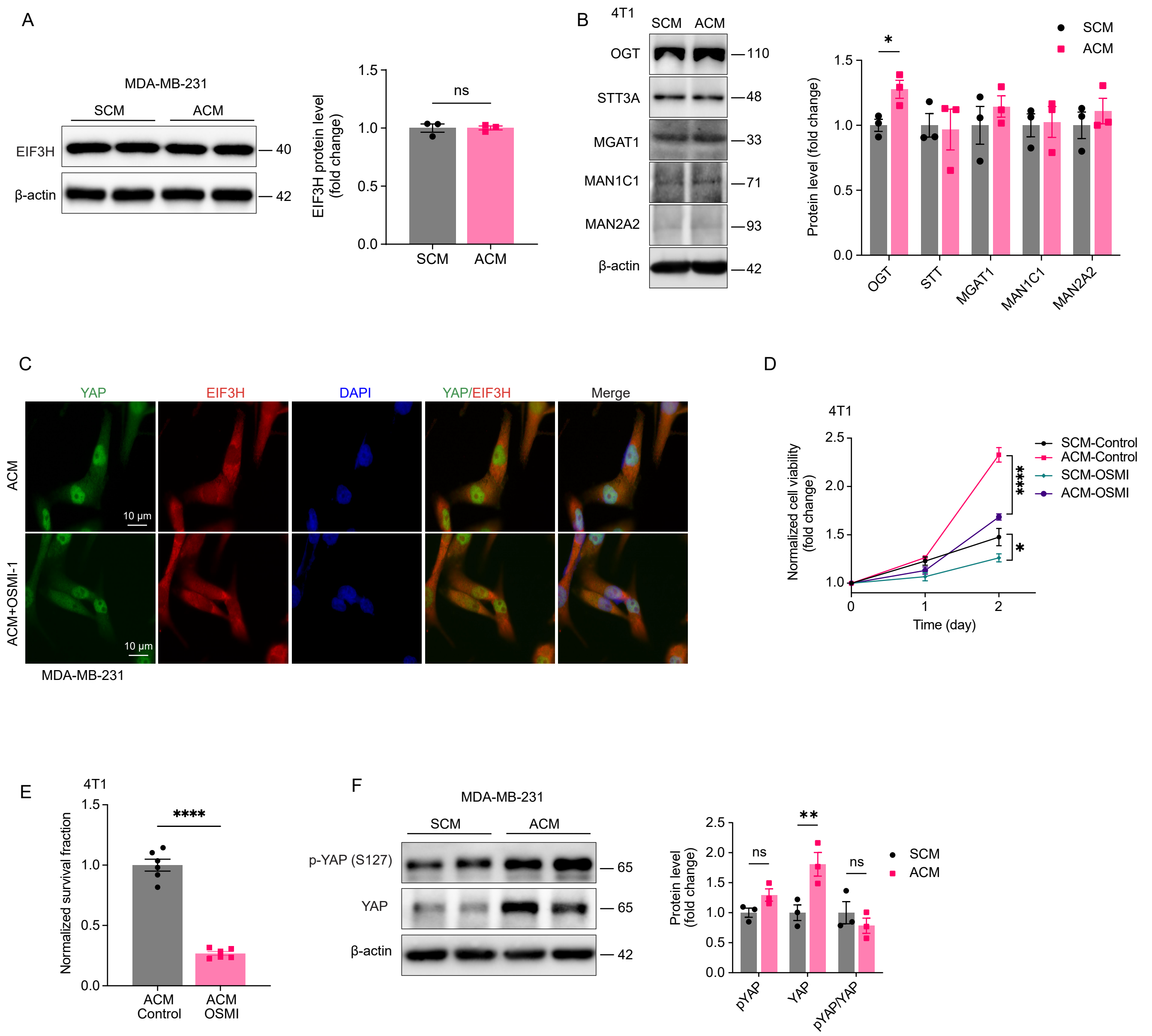
A
MDA-MB-231
SCM
ACM
EIF3H
— 40
β-actin
— 42
EIF3H protein level
(fold change)
ns
1.5
1.0
0.5
0.0
SCM
ACM
B
4T1
SCM
ACM
OGT
—110
STT3A
—48
MGAT1
—33
MAN1C1
—71
MAN2A2
—93
β-actin
—42
Protein level (fold change)
*
SCM
ACM
OGT
STT
MGAT1
MAN1C1
MAN2A2
C
YAP
EIF3H
DAPI
YAP/EIF3H
Merge
ACM
ACM+OSMI-1
10 μm
10 μm
MDA-MB-231
D
4T1
Normalized cell viability
(fold change)
SCM-Control
ACM-Control
SCM-OSMI
ACM-OSMI
****
*
Time (day)
E
4T1
Normalized survival fraction
****
ACM
Control
ACM
OSMI
F
MDA-MB-231
SCM
ACM
p-YAP (S127)
— 65
YAP
— 65
β-actin
— 42
Protein level
(fold change)
ns
**
ns
SCM
ACM
pYAP
YAP
pYAP/YAP

Figure S5

Figure S5. O-GlcNAcylation regulates EIF3H-YAP interaction and promotes adipocyte-induced YAP stabilization in TNBC cells.

(A) EIF3H protein level was determined and quantified in MDA-MB-231 cultured in ACM or SCM-conditioned medium (n=3). **(B)** The protein expression levels of multiple enzymes involved in both O-glycosylation and N-glycosylation pathways were determined in ACM or SCM treated 4T1 cells (n=3). **(C)** ACM-conditioned MDA-MB-231 cells were treated with OSMI-1. Colocalization between YAP and EIF3H and DAPI was measured by immunofluorescence staining, showing a decreased overlap of YAP (green) and EIF3H (red). **(D-E)** 4T1 cells were cultured using adipocytes conditioned medium and treated with OSMI-1 for 24 hours, CCK8 based cell proliferation (D) (n=4), and colony formation (E) (n=6) were determined. (**F**) YAP phosphorylation and the ratio to total YAP protein was determined in SCM or ACM treated MDA-MB-231 cells with the indicated antibodies (n=3). Data (mean ± SEM) are representative of at least three independent experiments. *$p < 0.05$, **$p < 0.01$, ***$p < 0.001$, and ****$p<0.0001$, by multiple unpaired t-test or two-way ANOVA followed with Tukey's multiple comparisons test.

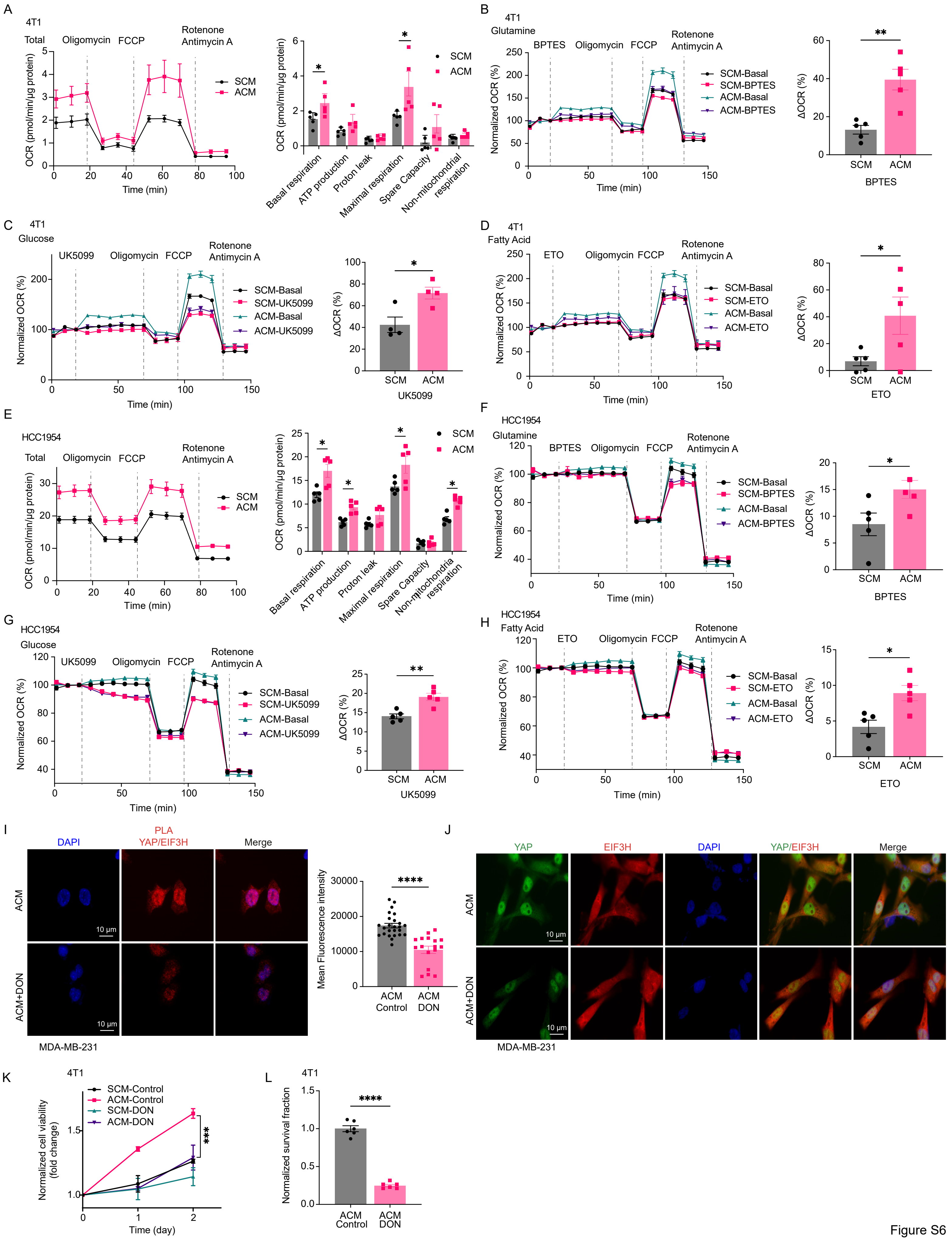
A
4T1
Total
Oligomycin
FCCP
Rotenone
Antimycin A
OCR (pmol/min/μg protein)
Time (min)
SCM
ACM
Basal respiration
ATP production
Proton leak
Maximal respiration
Spare Capacity
Non-mitochondrial respiration
B
Glutamine
BPTES
Normalized OCR (%)
SCM-Basal
SCM-BPTES
ACM-Basal
ACM-BPTES
ΔOCR (%)
C
Glucose
UK5099
SCM-UK5099
ACM-UK5099
D
Fatty Acid
ETO
SCM-ETO
ACM-ETO
E
HCC1954
F
G
H
I
DAPI
PLA
YAP/EIF3H
Merge
ACM
ACM+DON
10 μm
Mean Fluorescence intensity
ACM Control
ACM DON
MDA-MB-231
J
YAP
EIF3H
K
Normalized cell viability (fold change)
Time (day)
SCM-Control
ACM-Control
SCM-DON
ACM-DON
L
Normalized survival fraction

Figure S6

Figure S6. Adipocyte-induced metabolic dysregulation enhances YAP O-GlcNAcylation and accumulation, regulating EIF3H interaction and TNBC cell proliferation.

(A-D) The Mito stress (A) and mitochondrial substrates oxidation (B-D) were measured for ACM or SCM-treated 4T1 cells with a Seahorse XF24 Flux Analyzer. Basal respiration, ATP-linked respiration, maximal and reserve capacities, non-mitochondrial respiration for Mito stress and OCR change for substrates oxidation were determined (n=5). **(E-H)** The Mito stress (E) and mitochondrial substrates oxidation (F-H) were measured with the same method for ACM or SCM-treated HCC1954 cells (n=5). **(I)** ACM-conditioned MDA-MB-231 cells were treated with GFAT inhibitor DON and then incubated with mouse anti-YAP and rabbit anti-EIF3H antibodies. Duolink PLA was then performed and red dots indicate the interaction of the two proteins. The red fluorescence was also quantified (Control n=25, DON n=17). **(J)** ACM-conditioned MDA-MB-231 cells were treated with GFAT inhibitor DON. Colocalization between YAP and EIF3H and DAPI was measured by immunofluorescence staining, showing a decreased overlap of YAP (green) and EIF3H (red). **(K-L)** 4T1 cells were cultured using adipocytes conditioned medium and treated with DON for 24 hours, CCK8 based cell proliferation (K) (n=4), and colony formation (L) (n=6) were determined. Data (mean ± SEM) are representative of at least three independent experiments. *p < 0.05, **p < 0.01, ***p < 0.001, and ****p<0.0001, by multiple unpaired t-test or two-way ANOVA followed with Tukey's multiple comparisons test.

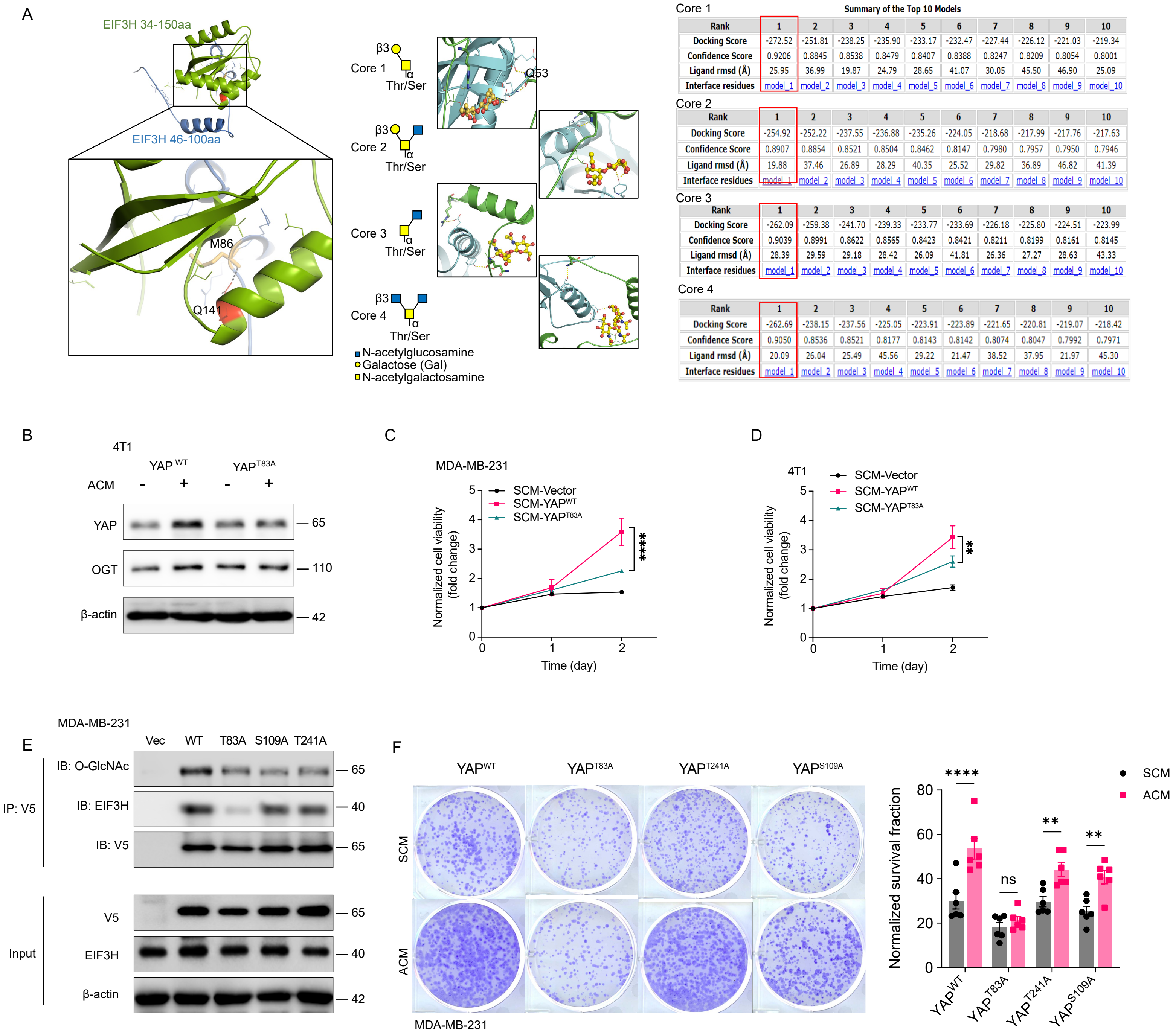

Core 1

Summary of the Top 10 Models

Rank	1	2	3	4	5	6	7	8	9	10
Docking Score	-272.52	-251.81	-238.25	-235.90	-233.17	-232.47	-227.44	-226.12	-221.03	-219.34
Confidence Score	0.9206	0.8845	0.8538	0.8479	0.8407	0.8388	0.8247	0.8209	0.8054	0.8001
Ligand rmsd (Å)	25.95	36.99	19.87	24.79	28.65	41.07	30.05	45.50	46.90	25.09
Interface residues	model_1	model_2	model_3	model_4	model_5	model_6	model_7	model_8	model_9	model_10

Core 2

Rank	1	2	3	4	5	6	7	8	9	10
Docking Score	-254.92	-252.22	-237.55	-236.88	-235.26	-224.05	-218.68	-217.99	-217.76	-217.63
Confidence Score	0.8907	0.8854	0.8521	0.8504	0.8462	0.8147	0.7980	0.7957	0.7950	0.7946
Ligand rmsd (Å)	19.88	37.46	26.89	28.29	40.35	25.52	29.82	36.89	46.82	41.39
Interface residues	model_1	model_2	model_3	model_4	model_5	model_6	model_7	model_8	model_9	model_10

Core 3

Rank	1	2	3	4	5	6	7	8	9	10
Docking Score	-262.09	-259.38	-241.70	-239.33	-233.77	-233.69	-226.18	-225.80	-224.51	-223.99
Confidence Score	0.9039	0.8991	0.8622	0.8565	0.8423	0.8421	0.8211	0.8199	0.8161	0.8145
Ligand rmsd (Å)	28.39	29.59	29.18	28.42	26.09	41.81	26.36	27.27	28.63	43.33
Interface residues	model_1	model_2	model_3	model_4	model_5	model_6	model_7	model_8	model_9	model_10

Core 4

Rank	1	2	3	4	5	6	7	8	9	10
Docking Score	-262.69	-238.15	-237.56	-225.05	-223.91	-223.89	-221.65	-220.81	-219.07	-218.42
Confidence Score	0.9050	0.8536	0.8521	0.8177	0.8143	0.8142	0.8074	0.8047	0.7992	0.7971
Ligand rmsd (Å)	20.09	26.04	25.49	45.56	29.22	21.47	38.52	37.95	21.97	45.30
Interface residues	model_1	model_2	model_3	model_4	model_5	model_6	model_7	model_8	model_9	model_10

Figure S7

Figure S7. Structural and functional insights into glycosylation and ubiquitylation interplay in adipocyte-induced YAP accumulation and EIF3H interaction.

(A) The structural model, derived from molecular docking simulations, elucidates the intermolecular interactions between the YAP (Q46-E100) TEAD domain and EIF3H (S34-L150) amino acid residues, particularly within the JAB/MP domain. The Left enlarged region shows predicted interacting residues, highlighting the specificity of the binding interface. Right panel, the investigation unveils the diversity in core glycan attachment at Thr83 of YAP, emphasizing its potential role in modulating the interaction landscape. The top 10 docking simulations and docking interaction energy were also calculated in the right panel. **(B)** OGT and YAP protein levels were determined in the SCM or ACM treated 4T1-YAP^{WT} and 4T1-YAP^{T83A} cells. **(C-D)** MDA-MB-231 cells (C) and 4T1 cells (D) with stably expressing V5-YAP^{WT} or V5-tagged YAP^{T83A} mutant, cultured with stromal-cell conditioned medium, CCK8-based cell proliferation was determined (n=4). **(E)** O-GlcNAcylation status and interaction with EIF3H of YAP was determined by co-immunoprecipitation of ectopic V5-tagged YAP^{WT} and YAP^{T83A}, YAP^{S109A}, YAP^{T241A} mutants in ACM-treated MDA-MB231 cells. **(F)** MDA-MB-231 cells with stably expressing V5-YAP^{WT} and V5-tagged YAP^{T83A}, YAP^{S109A}, YAP^{T241A} mutants were cultured using adipocytes conditioned medium or stromal-cell conditioned medium, colony formation was determined (n=6). Data (mean ± SEM) are representative of at least three independent experiments. *p < 0.05, **p < 0.01, ***p < 0.001, and ****p<0.0001, by multiple unpaired t-test or two-way ANOVA followed with Tukey's multiple comparisons test.

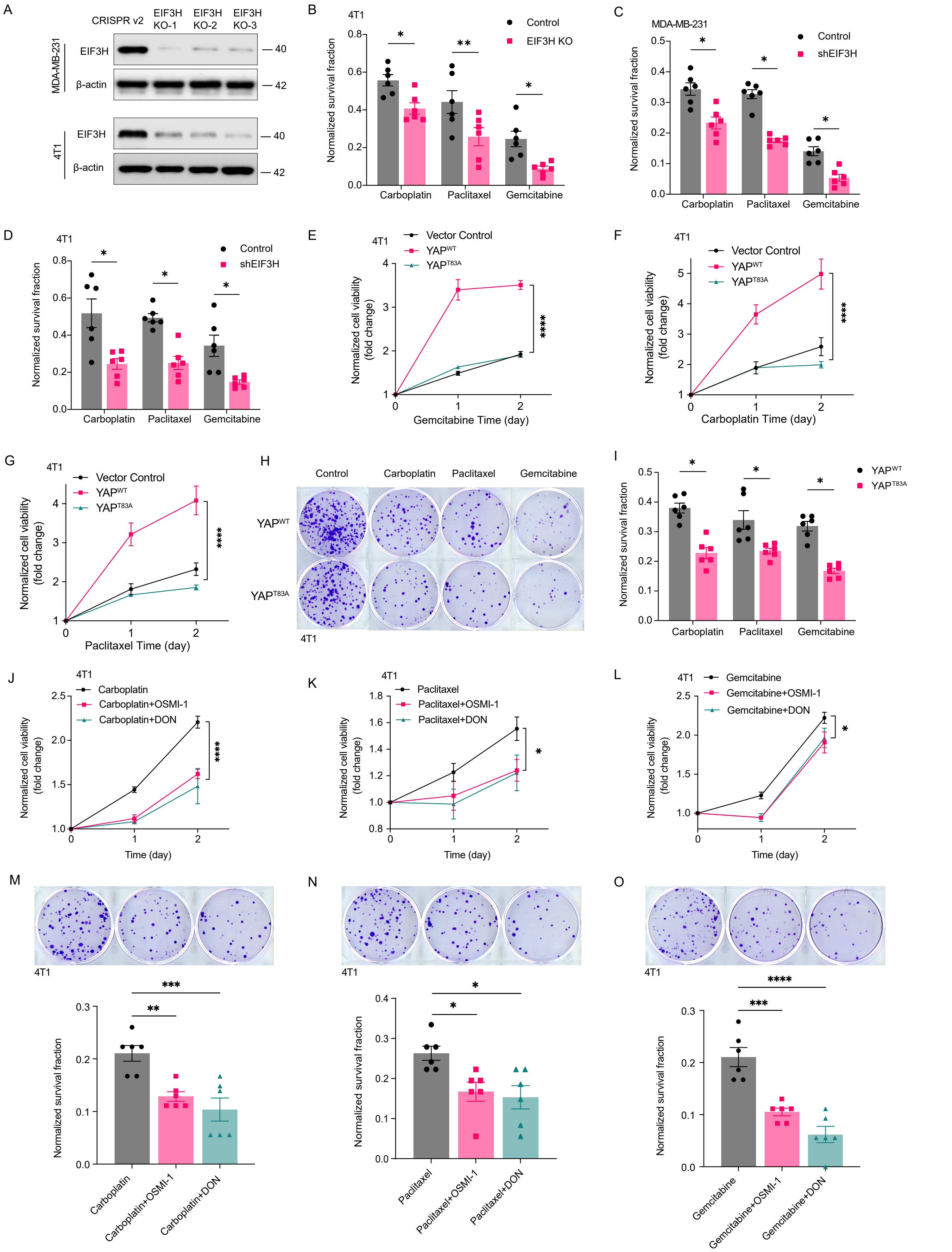
A
CRISPR v2
EIF3H KO-1
EIF3H KO-2
EIF3H KO-3
MDA-MB-231
EIF3H
β-actin
4T1
40
42
B
4T1
Control
EIF3H KO
Normalized survival fraction
Carboplatin
Paclitaxel
Gemcitabine
C
MDA-MB-231
Control
shEIF3H
D
4T1
E
Vector Control
YAPWT
YAPT83A
Normalized cell viability (fold change)
Gemcitabine Time (day)
F
Carboplatin Time (day)
G
Paclitaxel Time (day)
H
Control
Carboplatin
Paclitaxel
Gemcitabine
I
J
Carboplatin
Carboplatin+OSMI-1
Carboplatin+DON
Time (day)
K
Paclitaxel
Paclitaxel+OSMI-1
Paclitaxel+DON
L
Gemcitabine
Gemcitabine+OSMI-1
Gemcitabine+DON
M
N
O

Figure S8

Figure S8. Pharmacological targeting of the HBP-OGT-YAP axis restores chemosensitivity in adipocyte-conditioned TNBC cells.

(A) Three EIF3H knockout cell lines were established with different sgRNAs with CRISPR/Cas9 system. Empty vector CRISPRv2 used as control. **(B)** 4T1-Control and 4T1-EIF3H KO cells were cultured with ACM medium and exposed to 25 µg/ml Carboplatin, 5 µM Gemcitabine, and 500 nM Paclitaxel for 24h, colony survivals were determined (n=6). **(C-D)** shCon and shEIF3H stable cell lines of MDA-MB-231 (C) and 4T1 (D) cells were cultured with ACM medium and exposed to 25 µg/ml Carboplatin, 5 µM Gemcitabine, and 500 nM Paclitaxel for 24h, colony survivals were determined (n=6). **(E-I)** 4T1 vector control, 4T1-YAP^{WT} and 4T1-YAP^{T83A} cells were cultured with ACM medium and exposed to 25 µg/ml Carboplatin, 5 µM Gemcitabine, and 500 nM Paclitaxel for 24h, cell proliferation rate (E-G) (n=4) and clonogenic survival (H-I) (n=6) was determined and quantified. **(J-O)** CCK8 based cell proliferation rates and clonogenic survival were assessed after treating ACM cultured 4T1 with 1 µM OSMI-1 or DON, and additionally with 25 µg/ml Carboplatin (J and M), 500 nM Paclitaxel (K and N), or 5 µM Gemcitabine (L and O) for 24 hours ((J-L) n=4, (M-O) n=6). Data (mean ± SEM) are representative of at least three independent experiments. *p < 0.05, **p < 0.01, ***p < 0.001, and ****p<0.0001, by multiple unpaired t-test or two-way ANOVA followed with Tukey's multiple comparisons test.

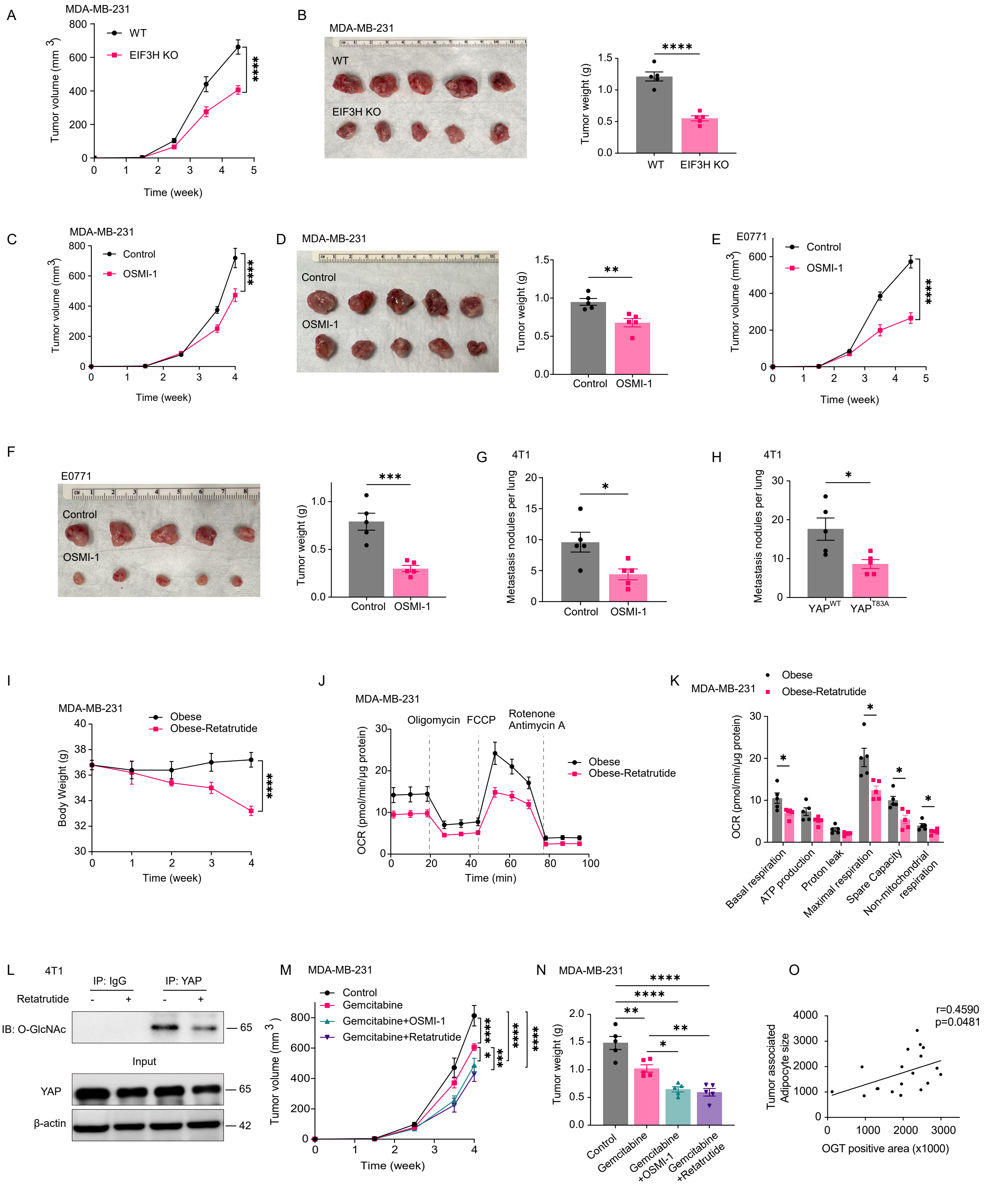
A
MDA-MB-231
WT
EIF3H KO
Tumor volume (mm3)
Time (week)
****
B
MDA-MB-231
WT
EIF3H KO
Tumor weight (g)
****
C
MDA-MB-231
Control
OSMI-1
Tumor volume (mm3)
Time (week)
****
D
MDA-MB-231
Control
OSMI-1
Tumor weight (g)
**
E
E0771
Control
OSMI-1
Tumor volume (mm3)
Time (week)
****
F
E0771
Control
OSMI-1
Tumor weight (g)
***
G
4T1
Metastasis nodules per lung
Control
OSMI-1
*
H
4T1
Metastasis nodules per lung
YAPWT
YAPT83A
*
I
MDA-MB-231
Obese
Obese-Retatrutide
Body Weight (g)
Time (week)
****
J
MDA-MB-231
Oligomycin
FCCP
Rotenone Antimycin A
Obese
Obese-Retatrutide
OCR (pmol/min/μg protein)
Time (min)
K
MDA-MB-231
Obese
Obese-Retatrutide
OCR (pmol/min/μg protein)
Basal respiration
ATP production
Proton leak
Maximal respiration
Spare Capacity
Non-mitochondrial respiration
L
4T1
IP: IgG
IP: YAP
Retatrutide
IB: O-GlcNAc
Input
YAP
β-actin
65
65
42
M
MDA-MB-231
Control
Gemcitabine
Gemcitabine+OSMI-1
Gemcitabine+Retatrutide
Tumor volume (mm3)
Time (week)
N
MDA-MB-231
Tumor weight (g)
Control
Gemcitabine
Gemcitabine +OSMI-1
Gemcitabine +Retatrutide
O
r=0.4590
p=0.0481
Tumor associated Adipocyte size
OGT positive area (x1000)

Figure S9

Figure S9. Retatrutide enhances chemosensitivity and reduces metastasis in obese TNBC models by targeting O-GlcNAcylation and metabolic dysregulation.

(A-B) WT and EIF3H KO MDA-MB-231 breast cancer cells were orthotopically injected into the right fourth mammary gland of the HFD-fed nude mice. The tumor growth curve was plotted (A), and tumor weight was measured at the end point (B) (n=5). **(C-F)** MDA-MB-231 (C-D) or E0771 (E-F) cells were orthotopically injected into the right fourth mammary fat pad in HFD-fed Nude or C57BL/6 mice and allowed to grow around 100 mm^3, followed by injection of OSMI-1 (5 mg/kg, i.p.) two times/week. Phosphate-buffered saline (PBS) was used in control mouse group. Tumor growth (C and E) and tumor weight (D and F) were plotted (n=5). **(G)** Lung metastasis model was generated by tail vein injection of $1x10^6$ 4T1 cells. OSMI-1 (5 mg/kg, i.p.) was administrated two times/week. Phosphate-buffered saline (PBS) was used in control mouse group. Lung metastases were allowed to develop for 4 weeks. Lung tissues were then collected, and the visible metastasis nodules were counted (n=5). **(H)** 4T1-YAP^{WT} and 4T1-YAP^{T83A} cells were injected via tail vein and lung metastasis was determined by the visible metastasis nodules (n=5). **(I-K)** MDA-MB-231 cells were orthotopically injected into the right fourth mammary fat pad in HFD-fed nude mice and allowed to grow around 100 mm^3, followed by injection of Retatrutide (5 mg/kg, i.p.) for two times/week. Mice body weights were monitored (I), and Retatrutide-treated tumors were harvested, digested and assayed for mitochondrial respiration (J-K) (n=5). **(L)** O-GlcNAcylation level of YAP was determined after Retatrutide treatment by coimmunoprecipitation. **(M-N)** MDA-MB-231 cells were orthotopically injected into the right fourth mammary fat pad in HFD fed nude mice and allowed to grow around 100 mm^3, followed by injection of Gemcitabine (5 mg/kg, i.p.) and Gemcitabine combined with OSMI-1 (5mg/kg, i.p.) or Retatrutide (5 mg/kg, i.p.) for two times/week. PBS were used in control groups. Tumor growth (M) was plotted, tumor weight was measured (N) (n=5). **(O)** The analysis of YAP and OGT in obese and non-obese human TNBC tissue sections using ImageJ shows a positive correlation between adipocyte size and OGT expression. Data (mean ± SEM) are representative of at least three independent experiments. *p

< 0.05, **$p < 0.01$, ***$p < 0.001$, and ****$p<0.0001$, by multiple unpaired t-test or two-way ANOVA followed with Tukey's multiple comparisons test.
